# Impact of a national collaborative project to improve the care of mechanically ventilated patients

**DOI:** 10.1371/journal.pone.0280744

**Published:** 2023-01-30

**Authors:** Yaseen M. Arabi, Zohair Al Aseri, Abdulmohsen Alsaawi, Ali M. Al Khathaami, Eman Al Qasim, Abdullah A. Alzahrani, Mohammed Al Qarni, Sheryl Ann I. Abdukahil, Hasan M. Al-Dorzi, Abdulaleem Alattasi, Yasser Mandourah, Tareef Y. Alaama, Mohammed K. Alabdulaali, Abdulrahman Alqahtani, Ahmad Shuaibi, Ali Al Qarni, Mufareh Alkatheri, Raed H. Al Hazme, Ramesh Kumar Vishwakarma, Omar Aldibasi, Mohammed Saeed Alshahrani, Ashraf Attia, Abdulrahman Alharthy, Ahmed Mady, Basheer Abdullah Abdelrahman, Huda Ahmad Mhawish, Hassan Ahmad Abdallah, Fahad Al-Hameed, Khalid Alghamdi, Adnan Alghamdi, Ghaleb A. Almekhlafi, Saleh Abdorabo Haider Qasim, Hussain Ali Al Haji, Mohammed Al Mutairi, Nabiha Tashkandi, Shatha Othman Alabbasi, Tariq Al Shehri, Emad Moftah, Basim Kalantan, Amal Matroud, Brintha Naidu, Salha Al Zayer, Victoria Burrows, Zayneb Said, Naseer Ahmed Soomro, Moawea Hesham Yousef, Ayman Abdulmonem Fattouh, Manar Aboelkhair Tahoon, Majdi Muhammad, Afifah Muslim Alruwili, Hossam Ahmed Al Hanafi, Pramodini B. Dandekar, Kamel Ibrahim, Mwafaq AlHomsi, Asma Rayan Al Harbi, Adel Saleem, Ejaz Masih, Nowayer Monawer Al Rashidi, Aslam Khan Amanatullah, Jaffar Al Mubarak, Amro Ali Abduljalil Al Radwan, Ali Al Hassan, Sadiyah Al Muoalad, Ammar Abdullah Alzahrani, Jamal Chalabi, Ahmad Qureshi, Maryam Al Ansari, Hend Sallam, Alyaa Elhazmi, Fawziah Alkhaldi, Abdulrauf Malibary, Abdullah Ababtain, Asad Latif, Sean M. Berenholtz, the Saudi Critical Care Trials Group

**Affiliations:** 1 Intensive Care Department, Ministry of National Guard Health Affairs, King Abdullah International Medical Research Center, King Saud Bin Abdulaziz University for Health Sciences, Riyadh, Kingdom of Saudi Arabia; 2 Department of Emergency, Department of Intensive Care, College of Medicine, King Saud University, Riyadh, Saudi Arabia; 3 Department of Medical Services, Ministry of National Guard Health Affairs, King Abdullah International Medical Research Center, King Saud Bin Abdulaziz University for Health Sciences, Riyadh, Saudi Arabia; 4 Quality and Patient Safety Department, Ministry of National Guard Health Affairs, King Abdullah International Medical Research Center, King Saud Bin Abdulaziz University for Health Sciences, Riyadh, Saudi Arabia; 5 Department of Military Medical Services, Ministry of Defense, Riyadh, Saudi Arabia; 6 Deputyship of Curative Services, Ministry of Health, Riyadh, Saudi Arabia; 7 Adaa Health Program, Ministry of Health, Riyadh, Saudi Arabia; 8 Executive Director of Medical Affairs Department, Ministry of Health, King Saud Medical City, Riyadh, Saudi Arabia; 9 Department of Medical Services, Ministry of National Guard Health Affairs, King Abdullah International Medical Research Center, King Saud Bin Abdulaziz University for Health Sciences, Dammam, Saudi Arabia; 10 Department of Medicine, Ministry of National Guard Health Affairs, King Abdullah International Medical Research Center, King Saud Bin Abdulaziz University for Health Sciences, Al Ahsa, Saudi Arabia; 11 Department of Health Informatics, Ministry of National Guard Health Affairs, King Abdullah International Medical Research Center, King Saud Bin Abdulaziz University for Health Sciences, Riyadh, Saudi Arabia; 12 Department of Biomedical Informatics, College of Osteopathic Medicine, Nova Southeastern University, Fort Lauderdale, Florida, United States of America; 13 Department of Bioinformatics and Biostatistics, Ministry of National Guard Health Affairs, King Abdullah International Medical Research Center, King Saud Bin Abdulaziz University for Health Sciences, Riyadh, Saudi Arabia; 14 Statistics Department, European Organisation for Research and Treatment of Cancer Headquarters, Brussels, Belgium; 15 Department of Critical Care, King Fahad Hospital of the University, Imam Abdulrahman Bin Faisal University, Al Khobar, Saudi Arabia; 16 Department of Intensive Care, King Saud Medical City, Riyadh, Saudi Arabia; 17 Department of Anesthesiology and Intensive Care, Tanta University Hospital, Tanta, Egypt; 18 Department of Intensive Care, Ministry of National Guard Health Affairs, King Abdullah International Medical Research Center, King Saud Bin Abdulaziz University for Health Sciences, Jeddah, Saudi Arabia; 19 Department of Intensive Care, King Faisal Specialist Hospital and Research Centre, Jeddah, Saudi Arabia; 20 Department of Intensive Care Services, Prince Sultan Military Medical City, Riyadh, Saudi Arabia; 21 Respiratory Services Department, Ministry of National Guard Health Affairs, King Abdullah International Medical Research Center, King Saud Bin Abdulaziz University for Health Sciences, Riyadh, Saudi Arabia; 22 Nursing Services, Ministry of National Guard Health Affairs, King Abdullah International Medical Research Center, King Saud Bin Abdulaziz University for Health Sciences, Riyadh, Saudi Arabia; 23 Rehabilitation Services Department, Ministry of National Guard Health Affairs, King Abdullah International Medical Research Center, King Saud Bin Abdulaziz University for Health Sciences, Riyadh, Saudi Arabia; 24 Department of Intensive Care, Arar Central Hospital, Arar, Saudi Arabia; 25 Department of Intensive Care, Alrass General Hospital, AlQassim, Saudi Arabia; 26 Department of Intensive Care, King Fahad Hospital, Al Baha, Saudi Arabia; 27 Department of Intensive Care, King Abdulaziz Specialist Hospital, Taif, Saudi Arabia; 28 Department of Intensive Care, Gurayat General Hospital, AlGurayat, Saudi Arabia; 29 Nursing Services, Prince Mohammed Bin Abdulaziz Hospital, Sakaka, Saudi Arabia; 30 Department of Intensive Care, King Salman Hospital, Riyadh, Saudi Arabia; 31 Department of Intensive Care, King Abdullah Hospital, Bisha, Saudi Arabia; 32 Department of Intensive Care, King Khalid General Hospital, Majmaah, Saudi Arabia; 33 Department of Intensive Care, Buraydah Central Hospital, AlQassim, Saudi Arabia; 34 Department of Intensive Care, King Fahad Specialist Hospital, AlQassim, Saudi Arabia; 35 Department of Intensive Care, King Faisal Hospital, Makkah, Saudi Arabia; 36 Department of Intensive Care, King Khaled Hospital, Tabuk, Saudi Arabia; 37 Department of Intensive Care, King Khalid Hospital, Hail, Saudi Arabia; 38 Respiratory Services, King Khalid General Hospital, Hafer Al Batin, Saudi Arabia; 39 Department of Intensive Care, Jubayl General Hospital, Jubayl, Saudi Arabia; 40 Department of Intensive Care, King Khalid Hospital, Najran, Saudi Arabia; 41 Nursing Services, Ministry of National Guard Health Affairs, King Abdullah International Medical Research Center, King Saud Bin Abdulaziz University for Health Sciences, Jeddah, Saudi Arabia; 42 Respiratory Services Department, Ministry of National Guard Health Affairs, King Abdullah International Medical Research Center, King Abdulaziz Medical City, King Saud Bin Abdulaziz University for Health Sciences, Jeddah, Saudi Arabia; 43 Department of Intensive Care, Ministry of National Guard Health Affairs, King Abdullah International Medical Research Center, King Saud Bin Abdulaziz University for Health Sciences, Al Ahsa, Saudi Arabia; 44 Department of Intensive Care, Ministry of National Guard Health Affairs, King Abdullah International Medical Research Center, King Saud Bin Abdulaziz University for Health Sciences, Madinah, Saudi Arabia; 45 Department of Intensive Care, Ministry of National Guard Health Affairs, King Abdullah International Medical Research Center, King Saud Bin Abdulaziz University for Health Sciences, Dammam, Saudi Arabia; 46 Department of Intensive Care, King Faisal Specialist Hospital and Research Centre, Riyadh, Saudi Arabia; 47 Nursing Services, King Faisal Specialist Hospital and Research Centre, Riyadh, Saudi Arabia; 48 Respiratory Services, Royal Commission Health Services Program, Jubayl, Saudi Arabia; 49 Department of Anesthesiology and Critical Care Medicine, Department of Surgery, The Johns Hopkins University School of Medicine, Baltimore, Maryland, United States of America; Mayo Clinic Minnesota, UNITED STATES

## Abstract

This prospective quasi-experimental study from the NASAM (National Approach to Standardize and Improve Mechanical Ventilation) collaborative assessed the impact of evidence-based practices including subglottic suctioning, daily assessment for spontaneous awakening trial (SAT), spontaneous breathing trial (SBT), head of bed elevation, and avoidance of neuromuscular blockers unless otherwise indicated. The study outcomes included VAE (primary) and intensive care unit (ICU) mortality. Changes in daily care process measures and outcomes were evaluated using repeated measures mixed modeling. The results were reported as incident rate ratio (IRR) for each additional month with 95% confidence interval (CI). A comprehensive program that included education on evidence-based practices for optimal care of mechanically ventilated patients with real-time benchmarking of daily care process measures to drive improvement in forty-two ICUs from 26 hospitals in Saudi Arabia (>27,000 days of observation). Compliance with subglottic suctioning, SAT and SBT increased monthly during the project by 3.5%, 2.1% and 1.9%, respectively (IRR 1.035, 95%CI 1.007–1.064, p = 0.0148; 1.021, 95% CI 1.010–1.032, p = 0.0003; and 1.019, 95%CI 1.009–1.029, p = 0.0001, respectively). The use of neuromuscular blockers decreased monthly by 2.5% (IRR 0.975, 95%CI 0.953–0.998, p = 0.0341). The compliance with head of bed elevation was high at baseline and did not change over time. Based on data for 83153 ventilator days, VAE rate was 15.2/1000 ventilator day (95%CI 12.6–18.1) at baseline and did not change during the project (IRR 1.019, 95%CI 0.985–1.053, p = 0.2812). Based on data for 8523 patients; the mortality was 30.4% (95%CI 27.4–33.6) at baseline, and decreased monthly during the project by 1.6% (IRR 0.984, 95%CI 0.973–0.996, p = 0.0067). A national quality improvement collaborative was associated with improvements in daily care processes. These changes were associated with a reduction in mortality but not VAEs.

**Registration** The study is registered in clinicaltrials.gov (NCT03790150).

## Introduction

Mechanical ventilation is a life-saving intervention but can be associated with several complications including ventilator-associated pneumonia (VAP), ventilator-induced lung injury, pulmonary edema, thromboembolism, delirium, and ICU-acquired weakness. The concept of ventilator-associated events (VAEs) has been introduced in 2013, with the premise of capturing harm not only from pneumonia but also from pulmonary edema, atelectasis, and acute respiratory distress syndrome [1]. The concept of VAEs has been validated in observational studies, where VAEs were associated with longer mechanical ventilation duration and ICU and hospital stay, and higher mortality [2–9]. However, interventional data on the preventability of VAEs are limited; there is inconsistent evidence that VAEs can be reduced by interventions, such as daily assessment for spontaneous awakening trial (SAT) and spontaneous breathing trial (SBT) that have been shown to improve other clinical outcomes in mechanically ventilated patients [10–12].

The objective of this study is to report whether a multicenter collaborative to improve the care of mechanically ventilated patients by implementing evidence-based practices, that include subglottic suctioning, daily assessment for SAT, SBT, head of bed elevation, and avoidance of neuromuscular blockers unless otherwise indicated, would reduce VAE rate and other outcomes including mortality.

## Materials and methods

### Setting and management

A pilot collaborative for improving the care of mechanically ventilated patients by implementing evidence-based practices was conducted between September 2015 and December 2016 in 15 ICUs in Saudi Arabia collaboratively with the Armstrong Institute for Patient Safety and Quality at the Johns Hopkins Hospital [13]. The pilot collaborative demonstrated the feasibility of a multicenter collaborative across ICUs in Saudi Arabia. As such, the National Approach to Standardize and Improve Mechanical Ventilation (NASAM) collaborative was planned as a national quality improvement project [14]. The premise was that implementing evidence-based practices would reduce VAE rate and other outcomes including mortality.

### Study design

**Fig 1:**
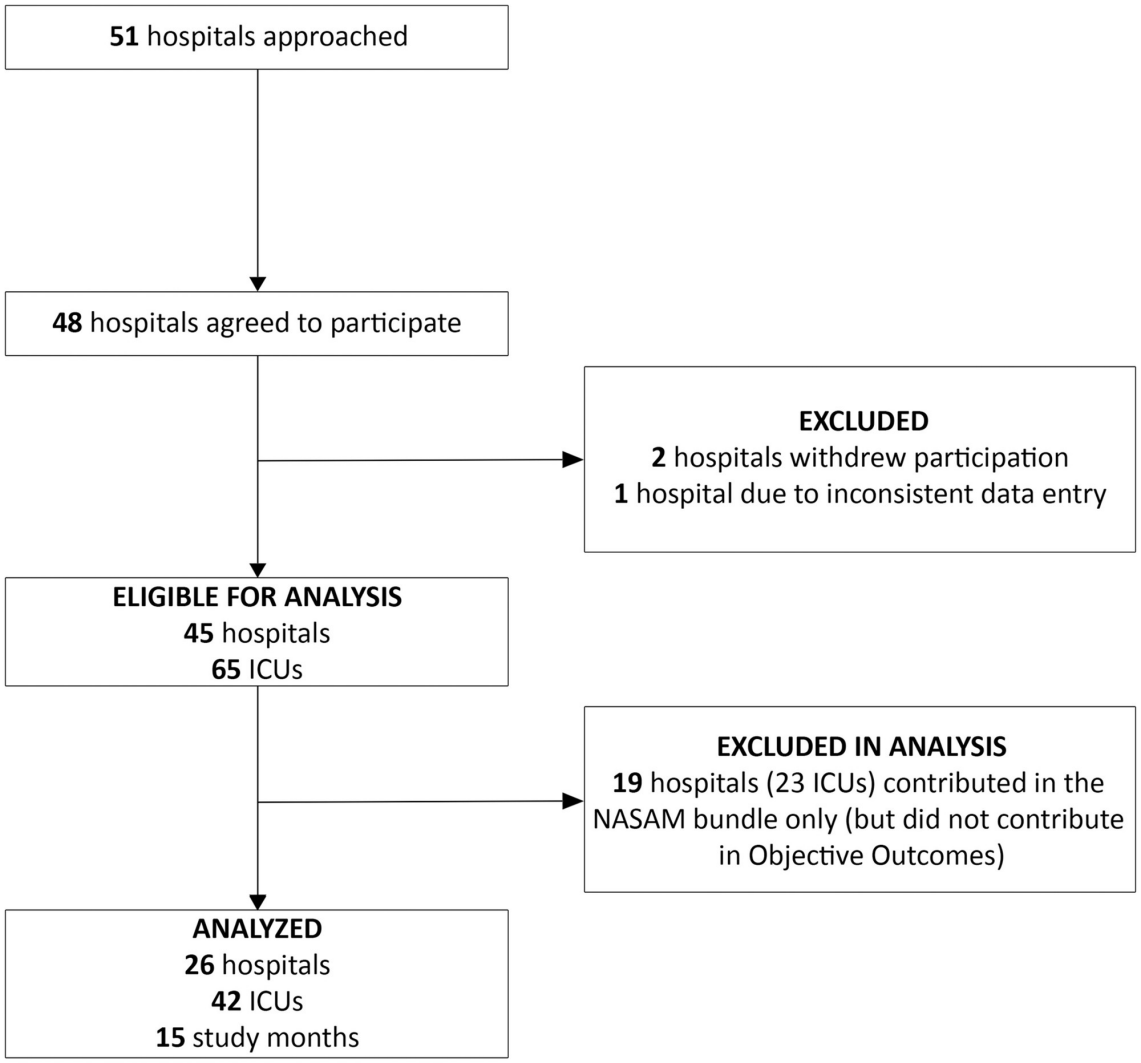
Study flow diagram.

The collaborative was designed as a prospective, multicenter, quasi-experimental study, in which compliance with daily care process measures and clinical outcomes for patients ventilated for ≥2 calendar days were monitored. The collaborative was launched in January 2019 and was planned as an 18-month project. During the coronavirus disease 2019 pandemic, several ICUs had logistic challenges continuing data entry. Therefore, the current report included data for 15 months (January 1, 2019 to March 31, 2020). The analysis population included ICUs that provided data on daily care process measures as well as clinical outcomes. (Fig 1) The Institutional Board Review of the Ministry of National Guard Health Affairs (RC 17/223) and all participating hospitals (S2 Table) reviewed the study and approved it in accordance with the ethical standards of the responsible committees on human experimentation and with the Helsinki Declaration of 1975. Given the nature of the study as a quality improvement project, and the lack of identifying patient level data, the need for informed consent was waived by the IRBs. The protocol was registered at clinicaltrials.gov (NCT03790150).

### The intervention

This collaborative aimed to improve the implementation of evidence-based practices, including the use of subglottic suctioning, SAT and SBT, head of bed elevation, and avoidance of neuromuscular blockers unless otherwise indicated [8, 15–17]. In addition, units had the option of implementing and monitoring interventions for early mobility and delirium assessment. As the number of units that participated in this module was small, the related data are not reported here.

This initiative was designed as a data-driven project, using an electronic platform for data entry that allowed real-time benchmarking with ICUs from the same hospital, same healthcare system, and the whole cohort. Data on daily care process measures were collected twice per week on all mechanically ventilated patients. The steering committee provided regular feedback on performance, which was relayed to the frontline teams to guide improvement efforts.

Before launching the project, a two-day workshop was conducted in November 2018 and attended by the multidisciplinary healthcare teams of participating ICUs. Additionally, webinars were conducted biweekly to provide training throughout the project, addressing best practices in patient safety and improving the care of mechanically ventilated patients (S3 Table). In addition, the webinars provided a platform for ICU teams to share their experiences and success stories. As accurate data collection was an integral part of this project, training sessions were provided regarding data definitions and data collection tools (S4 Table). In addition, various resources were made available through the project webpage including the webinar videos.

Participating ICUs formed unit-based multidisciplinary improvement teams consisting of physicians, nurses, respiratory therapists, and in some units, physiotherapists, and infection control practitioners, following the Comprehensive Unit-based Safety Program (CUSP) approach. This approach has been linked to large-scale reductions in healthcare-acquired infections [18–21], mortality [22], and associated costs [23]. These teams held unit-based meetings to review their unit’s progress, challenges, and ways to drive improvement. They reviewed reports of compliance with daily care processes and made action plans. They escalated issues that required further action to the hospital administration. CUSP teams from different hospitals presented their experiences in the biweekly webinars and shared established protocols. The collaborative engaged executives of healthcare systems and hospitals as a component of the CUSP framework to facilitate addressing system-level challenges [24].

### Measurement

#### Characteristics of participating units

We collected data on the characteristics of participating ICUs including the number of ICU beds and ICU staff (physicians, nurses, respiratory therapists, and physiotherapists or occupational therapists).

#### Measures of daily care processes

Compliance with any given daily care process was calculated for patients eligible for that daily care process, as defined in S4 Table. The measures of compliance with daily care processes included subglottic suctioning, head of bed elevation, SAT, percentage of ventilated patient days without sedation, SBT, percentage of actual RASS score -1 to +1, percentage of achieving RASS target and percentage of ventilated patients receiving neuromuscular blockers. Because the eligibility criteria differ for each daily care process, the denominators for calculating the compliance varied.

#### Primary outcome

The primary outcome was VAE rate as per the Centers for Disease Control and Prevention definitions [25]. We followed the hierarchy of definitions within VAE: a VAE that met the criteria for ventilator-associated condition [26] and infection-related ventilator-associated complication (IVAC) was reported as IVAC and a VAE that met the criteria for VAC, IVAC, and probable VAP (PVAP) was reported as PVAP [25]. The VAE rate was calculated as the number of VAEs (VACs +IVACs+ PVAPs) per 1000 ventilator days for each month.

Data were collected on VAEs by an assigned and trained respiratory therapist, nurse or infection control practitioner in each of the participating ICUs. The number of VAEs, including VAC, IVAC, and PVAP, was reported as a monthly aggregate. Outcome data were audited by a designated team, and inconsistencies and discrepancies were reviewed and resolved. VAE data were collected manually and then entered to the electronic data platform at the end of each month by all participating units. Range and sense checks were performed on all variables before commencing statistical analyses.

#### Secondary outcomes

Secondary outcomes including ICU mortality, duration of mechanical ventilation, and ICU length of stay were calculated from aggregate data reported from each unit as the number of deaths, the total number of mechanical ventilation days and total ICU days for mechanically ventilated patients admitted in a given month. Individual components of VAE (VAC, IVAC, and PVAP) were also secondary outcomes.

### Statistical analysis

Given the nature of this quality improvement project, there was no formal power calculation or a pre-specified sample size calculation. Because ICUs started at different time points, data were standardized so that the baseline study month was defined as the first month of data collection irrespective of the actual date. Descriptive standardized data on daily care process measures and outcomes were reported as unadjusted proportions for each month with an associated 95% confidence interval (CI). Line plots were generated for all study parameters.

#### Analysis of the primary outcome

In the primary outcome analysis (VAE rate), we used repeated measures mixed modeling (generalized linear and mixed model [GLMM]) with a Poisson distribution for the monthly number of VAEs, including an offset term for the number of ventilator days to account for variation due to lengths of time. The ICU and hospital were considered as random effects and the standardized study month as a fixed effect in the model. Results were expressed as incidence risk ratio (IRR) for each additional month in the project with 95%CI.

Diagnostic checking of residuals was performed for the parameter estimates. The over-dispersion of parameters was also assessed by calculating the ratio of the Pearson chi-square statistic and its degrees of freedom. If this ratio exceeded 1, it indicated that the variability in data was not properly modeled and that there was residual over-dispersion due to misspecification of the conditional distribution. When the Poisson regression model deviated from the assumption of equi-dispersion (average = variance), the negative binomial regression model was adopted. The model was selected as the best model with a unique covariance structure that produced the lowest Bayesian Information Criterion value. The covariance structures which were considered in the model were: first order of autocorrelation covariance structure, unstructured covariance structure, Toeplitz covariance structure and variance component structure. The random coefficients were modeled using G-side random effects and obtained the unit-specific estimates by defining the appropriate variance-covariance structure. The Newton-Raphson optimization technique with ridging option was used whenever the model failed to converge. We conducted a secondary analysis of VAE per 100 episodes of mechanical ventilation [8].

### Other outcomes and subgroup analysis

We conducted similar analyses to evaluate the change over time in the daily care process measures and secondary outcomes. The change over time was evaluated in predefined subgroups (low versus high baseline month compliance rates [categorized by the median of the cohort] of subglottic suctioning, SAT, and SBT, and ICUs in hospitals with >300 versus ≤300 beds), with an interaction term to assess the heterogeneity of treatment effect. The results of subgroup analyses were displayed as a forest plot. A p-value <0.05 was considered statistically significant and all analyses were performed using SAS 9.4 (SAS Institute, Cary NC).

## Results

### Participating ICUs

Forty-two ICUs contributed data on daily care process measures and outcome measures (S1 Fig). The characteristics of the ICUs are shown in S5 Table. The ICUs participated in the project for a median of 8 (interquartile range 7) months.

### Daily care process measures

**Fig 2:**
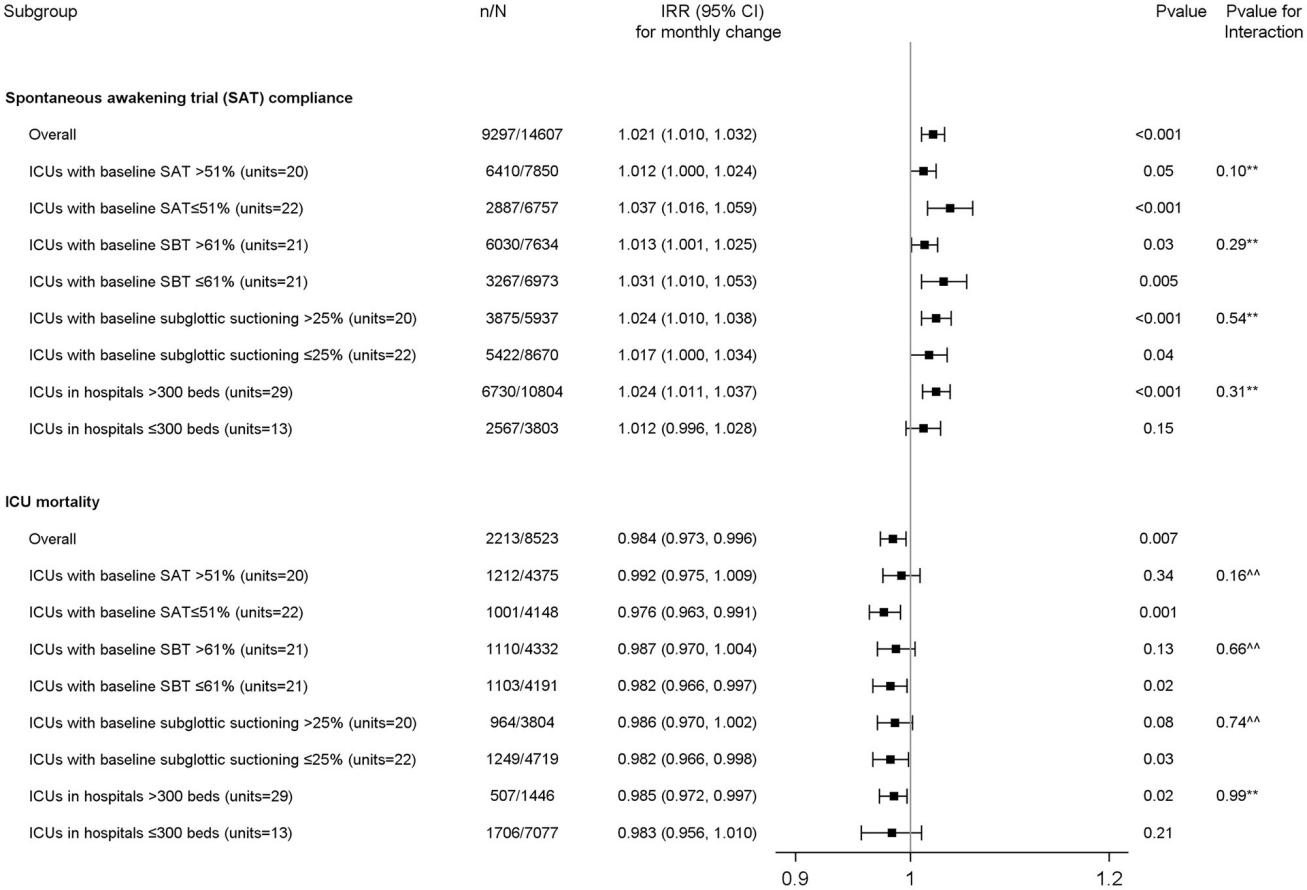
Forest plots for the change in spontaneous awakening trial (SAT) compliance and ICU mortality in different subgroups. The p-value for interaction is shown. Additional subgroup analyses are provided in supplement for subglottic suctioning, spontaneous breathing trial (SBT) and ventilator associated events (VAEs). Footnotes: ** The random effect Poisson regression was used to estimate incidence rate ratio after incorporating ICU unit and hospital as random effects.^^ The random effect negative binomial regression was used to estimate incidence rate ratio after incorporating ICU unit and hospital as random effects. IRR: Incidence rate ratio, CI: Confidence interval.

Data on subglottic suctioning compliance rate was available for 27840 eligible observations; the baseline month compliance rate was 32.4% (95%CI 30.2–34.7), which increased monthly by 3.5% (IRR 1.035, 95%CI 1.007–1.064, p = 0.0148) (Table 1, S6 Table and S2 Fig). Data on SAT compliance rate was available on 14607 eligible observations; the baseline month compliance rate was 48.7% (95%CI 45.3–52.1), which increased monthly by 2.1% (IRR 1.021, 95%CI 1.010–1.032, p = 0.0003, Fig 2). Data on SBT compliance rate were available on 21235 eligible observations; the baseline compliance rate was 53.1% (95%CI 50.4–55.7), which increased monthly by 1.9% (IRR 1.019, 95%CI 1.009–1.029, p = 0.0001). Data on the percentage of patients achieving RASS were available on 20017 eligible observations; the baseline compliance rate was 64% (95%CI 61.1–66.8), which increased monthly by 1.9% (IRR 1.019, 95%CI 1.003–1.036, p = 0.0220). Data on the percentage of the ventilated patients receiving neuromuscular blockers were available on 27966 eligible observations; the baseline compliance rate was 3.2% (95%CI 2.4–4.1), which decreased monthly by 2.5% (IRR 0.975, 95%CI 0.953–0.998, p = 0.0341). Data on head of bed elevation were available on 27752 eligible observations; the baseline compliance rate was 98.67% (95%CI 98.0–99.2), which did not change during the project (overall compliance rate 98.7%, 95%CI 98.5–98.8). An estimate of IRR for head of bed elevation could not be generated by GLMM due to poor fitting of the model, possibly because of limited variation across the study time points.

**Table 1:** Daily care process measures.

	Baseline month		Throughout the study duration		
	n compliant /N opportunities	% (95%CI)*	n compliant /N opportunities	IRR for change per month (95%CI)	p-value
Spontaneous awakening trial (SAT) compliance rate	417/856	48.7% (45.3, 52.1)	9297/14607	1.021 (1.010, 1.032)	0.0003†
Spontaneous breathing trial (SBT) compliance rate	719/1355	53.1% (50.4, 55.7)	13479/21235	1.019 (1.009, 1.029)	0.0001†
Subglottic suctioning compliance rate	556/1715	32.4% (30.2, 34.7)	10487/27840	1.035 (1.007, 1.064)	0.0148†
Percentage of achieving RASS target	708/1106	64% (61.1, 66.8)	14083/20017	1.019 (1.003, 1.036)	0.0220‡
Percentage of actual RASS score (-1 to 1)	475/1106	43% (40.0, 45.9)	7634/20017	0.996 (0.989, 1.008)	0.7906†
Percentage of ventilated patients receiving neuromuscular blocker	56/1761	3.2% (2.4, 4.1)	1181/27966	0.975 (0.953, 0.998)	0.0341†

*Unadjusted estimates are calculated from the first study month.

^†^ The random effect negative binomial regression was used to estimate incidence rate ratio after incorporating ICU unit and hospital as random effects.

^‡^ The random effect Poisson regression was used to estimate incidence rate ratio after incorporating ICU unit and hospital as random effects.

IRR: Incidence rate ratio, CI: Confidence interval, QR: Quarter range, RASS: Richmond Agitation Sedation Scale, SD: standard deviation

Data on head of bed elevation were available on 27752 eligible observations; the baseline compliance rate was 1709/1732, 98.67% (95% CI 98.0–99.2), which did not change during the project (overall compliance rate 27380/ 27752, 98.7%, 95% CI 98.5–98.8). An estimate of IRR for head of bed elevation could not be generated by GLMM due to poor fitting of the model, possibly because of limited variation across the study time.

### Outcomes

Data on VAE were available on 83153 mechanical ventilation days; the baseline VAE rate was 15.2% (95%CI 12.6–18.1), which did not change during the project (IRR 1.019, 95%CI 0.985–1.053, p = 0.2812) (Table 2 and S3 Fig). Secondary analysis showed that VAE rate per 100 episodes of mechanical ventilation did not change either (IRR 1.0147, 95%CI 0.979,1.052, p = 0.4268). There was also no change over time in the components of VAC, IVAC, or PVAP. Data on ICU mortality were available on 8523 patients; the baseline mortality rate was 30.4% (95%CI 27.4–33.6), which decreased monthly by 1.6% (IRR 0.984, 95%CI 0.973–0.996, p = 0.0067, Fig 2). There was no change in the duration of mechanical ventilation and ICU length of stay over time.

**Table 2:** Outcomes.

	Baseline month		Throughout the study duration		
	n/N	Event/1000 patient day (95% CI)*	n/N	IRR for change per month (95% CI)	p-value
Ventilator associated events (VAEs)	120/7916	15.2 (12.6, 18.1)	1350/83153	1.019 (0.985,1.053)	0.2812‡
VAC	55/7916	7.0 (5.2, 9.0)	741/83153	1.041 (1.011,1.073)	0.0079‡
IVAC	41/7916	5.2 (3.7, 7.0)	415/83153	0.974 (0.934,1.016)	0.2225‡
PVAP	24/7916	3 (1.9, 4.5)	194/83153	0.996 (0.942,1.052)	0.8783‡
	**n/N**	% (95% CI)*			
ICU mortality	259/851	30.4% (27.4, 33.6)	2213/8523	0.984 (0.973, 0.996)	0.0067‡
	**Days/patients**	**Mean (days)**	**Days/patients**		
Duration of mechanical ventilation	7095/851	8.3	78915/8523	0.994 (0.981,1.007)	0.335†
ICU Length of stay	9045/851	10.6	96871/8523	0.997 (0.984,1.011)	0.6773†

VAEs = VAC+IVAC+PVAC

* Unadjusted estimates are calculated from the first study month.

^†^ The random effect negative binomial regression was used to estimate incidence rate ratio after incorporating ICU unit and hospital as random effects.

^‡^ The random effect Poisson regression was used to estimate incidence rate ratio after incorporating ICU unit and hospital as random effects.

IRR: Incidence rate ratio, CI: Confidence interval, VAC: ventilator associated condition, IVAC: infection-related ventilator-associated complication, PVAP: possible ventilator-associated pneumonia

### Subgroup analysis

Subgroup analysis demonstrated that the improvement in daily care process measures and reduction in mortality was generally not different across different subgroups. VAE did not decrease in any subgroup (Fig 2, S3 and S4 Figs).

## Discussion

Our study demonstrates that an improvement collaborative for the care of mechanically ventilated patients was associated with improvement in daily care processes, including subglottic suctioning, (monthly increase of 3.5%), SAT (monthly increase of 2.1%), SBT (monthly increase of 1.9%) and the use of neuromuscular blockers (monthly decrease by 2.5%). These changes were not associated with a reduction in VAEs but were associated with a reduction in mortality (monthly decrease of 1.6%).

Our study also confirms what other studies have shown that the compliance with the daily ventilator care processes such as SAT and SBT, is often low [27, 28] but can be increased by an improvement [20, 27]. These daily care processes were among the interventions identified through the Delphi method as the most important and feasible to implement [29].

VAE surveillance has been designed to overcome the inaccuracy of VAP definition, enhance the detection of the complications of mechanical ventilation and serve as a possible quality measure. Using different surveillance methods, several studies have demonstrated that VAEs were associated with increased duration of mechanical ventilation, prolonged ICU and hospital length of stay, and increased mortality [2–9, 30–32]. However, these findings have not been consistent across studies. For example, a retrospective study of 1059 patients (15029 ventilator-days) found that VAE was not associated with mortality in multivariate analysis [33]. The important question is whether VAEs are amenable to improvement. Some studies demonstrated that VAEs are reduced with implementing evidence-based practices. A study from 56 ICUs at 38 hospitals demonstrated that improvement of evidence-based practices (including head-of-bed elevation, subglottic suctioning, oral care, chlorhexidine mouth care, and daily SAT and SBT) was associated with decrease in VAEs [27]. Another study showed that increases in SATs and SBTs were associated with decreases in mechanical ventilation duration and hospital stay, but not in VAE expressed in the standard approach (per ventilator day) [8]. When VAE risk was expressed as per episode of mechanical ventilation, a reduction was observed [8]. However, we did not observe reduction in VAE as expressed per 100 episodes of mechanical ventilation despite the improvement in daily care processes. Such a finding was observed in other studies. A retrospective, single-center study of 684 patients ventilated for >2 days, a ventilator bundle did not impact the incidence of VAC, IVAC, or VAP [34]. A prospective, single-center case-control study (n = 273 cases, n = 1092 controls) found that the compliance with VAP bundle was not associated with VAE [35]. On the contrary, increased compliance with chlorhexidine oral care was associated with increased VAE risk [35]. A randomized controlled trial (RCT) compared enhanced suctioning of the oropharynx every 4 hours with sham suctioning in ventilated patients and found similar VAE rates but reduced hospital length of stay [36].

There are several possible explanations for the lack of change in VAE rate despite improvement in evidence-based practices. First, there has been inconsistent evidence that VAEs are preventable [37]. Several studies highlighted the limitations of the VAE. A prospective cohort study found that VAE surveillance did not perform as well as VAP surveillance and had several undesirable characteristics [38]. The VAE constructs detected only one-third of VAP cases, and most VAE cases did not have VAP [38]. VAE rates were reduced by 93% by manipulation of ventilator management protocols [38]. A study that evaluated electronic VAE surveillance also showed low concordance with prospective VAP surveillance [9]. Other studies also demonstrated that the agreement between VAE and VAP was poor [30, 31, 33, 39], with VAE having low sensitivity to detect VAP and mostly representing non-preventable events [38, 40–43]. In addition, VAE is frequently due to noninfectious causes such as pulmonary edema, atelectasis, pneumothorax or non-cardiogenic edema. Significant risk factors for VAE include positive daily fluid balances ≥50 ml, long-acting analgesic/sedative, and daily gastric retention ≥200 ml [44–46]. Not all these factors are addressed in the commonly-used ventilator bundles and so an effect from these bundles may not be expected [37]. No study has thus far tested a VAE prevention bundle that addresses all potentially-modifiable VAE risk factors [47].

Our study demonstrated that the quality improvement collaborative was associated with a reduction in mortality. Several studies have evaluated the individual and collective impact of ventilator bundle elements. A systematic review of 13 studies showed that the implementation of a ventilator bundle significantly reduced mortality (odds ratio 0.90, 95%CI 0.84–0.97) [48]. Another systematic review of 45 randomized clinical trials involving 5493 participants showed that daily sedation interruption significantly reduced mechanical ventilation duration, ICU stay, sedation duration, tracheostomy and VAP risks [49]. In another improvement collaborative, the implementation of the ABCDEF bundle was associated with lower likelihood of hospital death within 7 days [17]. The reduction on mortality in our study was independent of VAE. This finding was observed in other studies [34] which indicates that improvements in daily care processes can result in clinical outcome improvement that is not measured by VAE. The lack of change in ICU LOS and mechanical ventilation duration could be attributed to the reduced mortality, as these are considered to be competing outcomes.

The strengths of this study include the large sample size and the multicenter participation. The statistical model, which is suitable for multi-level data, accounted for the heterogeneity across ICUs and hospitals. The standardization of the study month allowed for robust interpretation of the effect of the intervention over time. We used several approaches for improvement, including education, benchmarking and feedback. The use of multiple approaches is important because education alone has been shown to lead to small improvements. Limitations include the non-randomized design; raising the possibility that the association between the implementation of daily care processes and mortality may be due to unmeasured confounders, including changes in practice or measurement. Because of the COVID-19 pandemic, the study was shortened from a planned 18-month to 15-month period, resulting in a median center contribution of 8 months. A longer intervention would possibly have led to more improvement. Further work is needed to evaluate approaches for sustainability, including for example, automation of data collection and reporting. Given the design of the study, patient-level detailed data were not collected, therefore it was not possible to adjust for patient characteristics or severity of illness. We did not assess inter-rater differences in assessing compliance or in determining VAEs. Finally, we did not assess the relative effectiveness of the separate components of the intervention. Nevertheless, this collaborative initiative is probably among the largest initiatives that used VAE constructs as the primary outcome for improving the care of mechanically ventilated patients outside North America. These findings suggest the need for randomized clinical trials to examine the effect of this bundle of interventions on VAEs and mortality among mechanically ventilated patients.

## Conclusions

This quality improvement collaborative demonstrated that implementing evidence-based practices for the care of mechanically ventilated patients was associated with improvement in daily care process measures, including compliance rates with subglottic suctioning, SAT and SBT. Despite no decrease in VAEs, it was associated with reduction in mortality.

## Supporting information

S1 Table The Saudi Critical Care Trials Group. (PDF)

S2 Table List of participating sites and ethics committee approvals. (PDF)

S3 Table List of interventions. (PDF)

S4 Table Data measures definitions. (PDF)

S5 Table Characteristics of participating sites. IQR: interquartile range.
(PDF)

S6 Table Distribution of compliance, non-compliance and contraindications (with reasons) to the interventions. (PDF)

S1 Fig Map of Saudi Arabia with distribution of participating ICUs. (PDF)

S2 Fig Change of daily process measures over the study period among the whole cohort and in subgroups of ICUs with baseline spontaneous trial (SAT) compliance of >50% and ≤50. (PDF)

S3 Fig Change of ICU mortality and ventilator-associated events (VAE) over the study period among the whole cohort and in subgroups of ICUs with baseline spontaneous trial (SAT) compliance of >50% and ≤50%. (PDF)

S4 Fig Forest plots for the change in subglottic suctioning, spontaneous breathing trial (SBT) and ventilator associated events (VAEs) in different subgroups. ** The random effect Poisson regression was used to estimate incidence rate ratio after incorporating ICU unit and hospital as random effects. ^^ The random effect negative binomial regression was used to estimate incidence rate ratio after incorporating ICU unit and hospital as random effects. IRR: Incidence rate ratio, CI: Confidence interval.
(PDF)

S1 File Minimal underlying data. (XLSX)

S2 File (PDF)

S1 Checklist CONSORT 2010 checklist of information to include when reporting a randomised trial*. (DOC)
