# Supplementary material for: Impact of a national collaborative project to improve the care of mechanically ventilated patients
Source: PLoS One. 2023 Jan 30;18(1):e0280744. doi: 10.1371/journal.pone.0280744 (PMC9886257; doi:10.1371/journal.pone.0280744)
Supplement: S1 Table — (PDF) [file pone.0280744.s001.pdf]

NASAM management, writing committees, and site collaborators (Saudi Critical Care Trials Group)

**Management Committee**	Yaseen M. **Arabi**<br>Zohair **Al Aseri**<br>Tareef **Alaama**<br>Abdulrahman **Alqahtani**<br>Eman **Al Qasim**<br>Abdullah **Alzahrani**<br>Mohammed **Al Qarni**<br>Sheryl Ann **Abdukahil**<br>Abdulmohsen **Saawi**<br>Abdulaleem **Alattasi**<br>Yasser **Mandourah**<br>Hasan M **Al-Dorzi**<br>Ahmad **Shuaibi**<br>Ali **Khathami**<br>Ali **Alqarni**<br>Mufareh **Alkatheri**<br>Raed H **Al-Hazme**<br>Sean M **Berenholtz**<br>Asad **Latif**
**Writing Committee**	Yaseen M. **Arabi**<br>Eman **Al Qasim**<br>Sheryl Ann **Abdukahil**<br>Ramesh Kumar **Vishwakarma**<br>Sean M **Berenholtz**<br>Asad **Latif**<br>Saleh Abdorabo Haider **Qasim**
**Collaborators (Saudi Critical Care Trials Group)**	
Ministry of National Guard Health Affairs<br>King Saud bin Abdulaziz University for Health Sciences and King Abdullah International<br>Medical Research Center, Riyadh, Saudi Arabia	Yaseen M. **Arabi**<br>*(SCCTG Lead yaseenarabi@yahoo.com)*<br>Eman **Al Qasim**<br>Sheryl Ann **Abdukahil**<br>Abdullah **Alzahrani**<br>Mohammed **Al Qarni**<br>Ramesh Kumar **Vishwakarma**<br>Abdulmohsen **Saawi**<br>Abdulaleem **Alattasi**<br>Hasan M **Al-Dorzi**<br>Ali **Khathami**<br>Mufareh **Alkatheri**<br>Raed H **Al Hazme**<br>Mohamed S **Al-Moamary**<br>Saleh Abdorabo Haider **Qasim**<br>Hussain Ali **Al Haji**<br>Mohammed **Al Mutairi**<br>Nabiha **Tashkandi**<br>Shatha Othman **Alabbasi**<br>Tariq **Al Shehri**

	Emad **Moftah**<br>Basim **Kalantan**<br>Waeal **Al Anazi**<br>Amal **Al Matroud**<br>Brintha **Naidu**<br>Salha **Al Zayer**<br>Victoria **Burrows**<br>Zayneb **Said**<br>Fahima **Alsomali**<br>Musharaf **Sadat**<br>Felwa **bin Humaid**<br>Afrah **AlHarbi**<br>Abdullah **Almutairi**<br>Jenan **Al Jaithen**<br>Kholoud Khalid **Almufarrij**<br>John Dudley **Alchin**<br>Saeed **Obbid**<br>Fuad **Al Ghamdi**<br>Bander Faleh **Alotaibi**<br>Abdulkareem Farhan **Alanazi**<br>Muhammad Hassan **Alsomali**<br>Muhammad Obaid **Alshammari**<br>Ali Nasser **Al Aliani**<br>Ahmed **Majrashi**<br>Mohammed **Al Shahrani**<br>Jalal **Al Qahtani**<br>Khalaf **Al Anazi**<br>Majid **Al Zahrani**<br>Omar **Al Faris**<br>Nawaf **Al Anaizi**<br>Sami **Al Osaimi**<br>Mohammed **Obaidi**<br>Mohammed Shaim **Al Anazi**<br>Othman **Abahussein**<br>Abdullah Khalil **Al Anazi**<br>Naif **Mohaimeed**<br>Faisal **Al Baseet**<br>Muhammad **Aziz**<br>Haseenah **Khan**<br>Mohammed **Melhem**<br>Cathy **Gonzales**<br>Aluce **Ally**<br>Nadyah **Al Anizy**<br>Viccie **Ngobeni**<br>Manal **Hearsi**<br>Siham **Saeed**<br>Vilasini **Nair**<br>Saja **Al Marhoun**<br>Mohamed S **Al-Moamary**<br>Abdulaziz Saad **Al-Dawood**<br>Salim Alwi **Baharoon**

	Saad Mana **Al-Qahtani**<br>Haytham **Tlayjeh**<br>Basel Adnan **Al Raiy**<br>Tarek **Al-Dabbagh**<br>Raymond Mahindra **Khan**<br>Farhan Zayed **Al Enezi**<br>Alawi Sharaf **Alsaeedi**
King Saud Medical City - Riyadh, Saudi Arabia	Abdulrahman **Alharthy**<br>Ahmed **Mady**<br>Basheer Abdullah **Abdelrahman**<br>Huda Ahmad **Mhawish**<br>Hassan Ahmad **Abdallah**<br>Mohamed A. **Adam**<br>Tasmiya **Asad**<br>Ayman Hammad **Al Salmi**<br>Daood Saied **Asad**<br>Tri **Widyawati**<br>Kriz Louie **Odchigue**<br>Karthika **Janakiraman**<br>Karen Joyce **Calamba**<br>Chitradevi **Rajaram**<br>Booma **Thirognamam**<br>Hala Abdalla **Amer**<br>Emmylou Nunez **Mahinay**<br>Mohammad Hosni **Badawi**<br>Basel Hamid **Almuabbadi**
Arar Central Hospital - Arar, Saudi Arabia	Naseer Ahmed **Soomro**<br>Mahmoud **Mahdy**<br>Muqtadar Hajrani **Jani**<br>Muhammed Adeel **Mughal**<br>Abdul **Qhayyum**<br>Muhammad **Usman**<br>Iftikhar **Aslam**<br>Atta Muhammad **Jamali**<br>Suha **Mamoun**<br>Yasmeen Zal **Al Anizi**<br>Sujata **Natarajan**<br>Latifa Safouq **Al Anizi**
Alrass general hospital - AlQassim, Saudi Arabia	Moawea Hesham **Yousef**<br>Ayman Ibrahim **Abd elhakam**<br>Saad Abdullah **Almutairi**<br>Magdalena **Barrientos**<br>Khaled abdelfattah **Rashwan**
King Fahad Hospital - Al Baha, Saudi Arabia	Ayman Abdulmonem **Fattouh**
King Abdulaziz Specialist Hospital - Taif, Saudi Arabia	Manar Aboelkhair **Tahoon**<br>Thar bidar **Mohammed**<br>Mohammed Ibrahim H. **El Dosoqi**<br>Hayat Mohammaed **Al Qurashi**<br>Seham **Al Zain**
Gurayat General Hospital - AlGurayat, Saudi Arabia	Majdi **Muhammad**

	Melody **Manalo**<br>Carmela **Quijano**<br>Enrico **Caliguia**<br>Analyn **Tani**<br>Naser **Alenizy**
Prince Mohammed Bin Abdulaziz Hospital - Skakka, Saudi Arabia	Afifah Muslim **Alruwili**<br>Abdelrahman Mohammed **Shoieb**<br>Nabeela K. **Asri**<br>Taghreed **Alrowili**<br>Khalda **Alshammari**<br>Zainb **Abdelgadir**<br>Mastoora Marai **Al Habbad**<br>Farha **Arabi**<br>Sali **Al Nagem**
King Salman Hospital - Riyadh, Saudi Arabia	Hossam Ahmed **Al Hanafi**<br>Abdulhaleem Omer **Sadeeq**<br>Hajer Askar **Al Askar**<br>Halah Mohammed **Ghalby**<br>Bader Ahmed **Alfowzan**
King Abdullah Hospital - Bisha, Saudi Arabia	Pramodini **Dandeker**<br>Akin **Akinyemi**<br>Khaled **Haidi**<br>Moaid **al-Salloowm**<br>Ambreen **Akram**<br>Corrin **Perciano**<br>Charlotte **adante**<br>Joy **Nwokoro**<br>Mutlay **Alswat**
King Khalid General Hospital - Majmaah, Saudi Arabia	Kamel **Ibrahim**<br>Khalid **Mahmoud**<br>Mohammed **Mekawi**
Buraydah Central Hospital - AlQassim, Saudi Arabia	Mwafaq **AlHomsi**<br>Mahmoud **El Shennawy**
King Fahad Specialist Hospital - AlQassim, Saudi Arabia	Asma Rayan **Al Harbi**<br>Mohamed **Elsherbiny**<br>Salah Salem **Abou-Seif**<br>Jocelyn Francusco
King Faisal Hospital - Makkah, Saudi Arabia	Adel **Saleem**<br>Amal Nabil **Kaki**<br>Nadia Ateeq **AlMatani**
King Khaled Hospital - Tabuk, Saudi Arabia	Ejaz **Masih**<br>Nowayer Monawer **Al- Rashidi**<br>Mohammed Samer **Hajeh**<br>Wejdan Adam Ahmed **Ajjak**<br>Reham Mohamed Abdelmoniem **Ibrahim**<br>Mai Mohamed Bahgat **Ahmed**<br>Neama Shabaan **Mohammed**<br>Al- Naif Umda **Askali**<br>Leonilo Gumabon **Dela Cruz**

King Khalid Hospital - Hail, Saudi Arabia	Aslam Khan **Amanatullah**<br>Abdullah **Al Zarooq**<br>Nuran Mohamed **Al Hamad**<br>Layla Lafe Rashed **Alharby**<br>Fahad Rashid **Alhathal**
King Khalid General Hospital - Hafer Al Batin, Saudi Arabia	Jaffar **Al Mubarak**<br>Yusuf Abu **AlHasan**<br>Hanan **Al Mutairi**
Jubayl General Hospital - Jubayl, Saudi Arabia	Amro Ali Abduljalil **Al Radwan**<br>Saad **Al Dossari**<br>Saleh Abdalkarim **Alnass**<br>Sahar Mubarak **Al-Dowlab**<br>Fatima Abdullah **AlSadeq**<br>Yusf **Athweeny**<br>Basheer **Al Bahrani**<br>Deyaa **Radwan**
King Khalid Hospital - Najran, Saudi Arabia	Ali Alhassan
Qatif Central Hospital - Qatif, Saudi Arabia	Abdullah **Al Ramadan**<br>Riyda **Al Mousa**<br>Zaki **Al Zaher**<br>Mazen **Al Zayer**<br>Zahra **Al Manasef**<br>Bassam **Madan**<br>Zainab **Al Yousef**<br>Hassan Abdulbari **Almomen**
Al Noor Specialist Hospital - Makkah, Saudi Arabia	Ramzan Meeral **Sayed**<br>Qasim **Al Khateeb**<br>Mohannad Awadh **Al Harthi**<br>Siraj TasleeM **Jaishi**<br>Kelthom **Zakaria**
Prince Mohammed Bin Abdulaziz Hospital - Riyadh, Saudi Arabia	DaifAllah Saud D. **Al Otaibi**<br>Ayad Y.**Asairi**<br>Muhammad Ahmad **Almansour**<br>Munirah Musaed **Albeshi**
King Fahad Hospital - AlMadinah, Saudi Arabia	Ayman **Kharaba**<br>Abdulaziz **Al Harbi**<br>Abdulrahman Fahad **Al Arewi**<br>Kholud **Al Moulid**<br>Kholud Abdullah **Aljohani**<br>Moneraa **AlEnzi**
King Fahad Specialist Hospital - Tabouk, Saudi Arabia	Ayman **Hashem**<br>Elazebith **Matu**<br>Reem **Balawi**
Dammam Medical Complex - Dammam, Saudi Arabia	Dr. Dia Nasser **Al Nughaimish**<br>Fayez Yahya **AlEssa**<br>Fatimah Saeed **AlMarhoon**<br>Salma Kamel **AlHomoud**<br>Haoura Ali **AlMohammed Saleh**<br>Abdullah Saeed **AlZaki**<br>Rawan **AlSanea**

	Mohammed **Alabdrabalnabi**<br>Dia Nasser **AlNughaimish**<br>Ibrahim El Sayed **El Alfi**
King Faisal Medical Complex - Taif, Saudi Arabia	Nadia Mohammad **Samad**<br>OsamaSodki **Yassin**<br>Muhammad Rehan **Malik**
Prince Saud bin Jalawi - Al Hasa, Saudi Arabia	Saud Saleh **Alghannam**<br>Khalthom **Al Boali**<br>Akeela Mohd **Alfaiz**<br>Abdulelah Ali **Bin Saleh**<br>Hanan Abdulmohsen **Al Sultan**
King Abdulaziz Medical City, Ministry of National Guard Health Affairs, Jeddah, Saudi Arabia	Fahad **Al-Hameed**<br>Sadiyah **Al Muoalad**<br>Ammar Abdullah **Alzahrani**<br>Ohoud Mohammed **Aloraabi**<br>Mayadah Mustafa **Alhabshe**<br>Riyadh Ali **Alshehri**<br>Hashim Mansour **Alsharif**<br>Chula Anak **Edward**<br>Maher Mancera **Patasin**<br>Hadia **Tabsh**<br>Elham **Bukhari**<br>Basem **Banat**<br>Omar **Abu Skout**<br>Ramalakshmi **Ramadu**<br>Chula **Edward**<br>Maher **Patasin**
King Abdulaziz Hospital, Ministry of National Guard Health Affairs, Alhasa, Saudi Arabia	Jamal **Chalabi**<br>Majed **Zowaidi**<br>Dominica **Rose Daniel**<br>Layla **Al-Meer**
Prince Mohammed bin Abdulaziz, Ministry of National Guard Health Affairs, Madinah, Saudi Arabia	Ahmad Shahzad **Qureshi**<br>Ebrahim **Jaber**<br>Zaam **Al Otaibi**<br>Za'am Nejer **Alotaibi**<br>Adnan Hassan **Alhowaiti**<br>Feras Khalid **Melebari**<br>Abdulrahim Marzouq **Alalwi**<br>Majed Mohammad **Agdy**<br>Ahmad Muraishid **Almutairi**
Imam Abdulrahman Al Faisal Hospital, Ministry of National Guard Health Affairs, Dammam, Saudi Arabia	Maryam Al ansari<br>Manal Alahmari
King Faisal Specialist Hospital & Research Centre, Riyadh, Saudi Arabia	Hend **Sallam**<br>Alyaa **Elhazmi**<br>Fawziah **Alkhaldi**<br>Khalid **Maghrabi**<br>Mehareen **Bano**<br>Reji **Jose**<br>Bashair **Almegren**<br>Eyad **Aljulayfi**

	Nor Safrinah **Hanifah**<br>Sanaa **Alenazi**<br>Rahaf Saud **Alrashoud**<br>Shatha **Barajaa**<br>Ruba **Dmairi**<br>Fai **Alanazi**
King Faisal Specialist Hospital & Research Centre, Jeddah, Saudi Arabia	Khalid **Alghamdi**<br>Ismael **Qushmaq**<br>Lama Khaled **Hefni**<br>Rana **Albatati**<br>Samer **Althaqafi**<br>Maram **Albalawi**<br>Alia **Darwish**
Prince Sultan Military Medical City, Riyadh, Saudi Arabia	Adnan **Al Ghamdi**<br>Ghaleb **Almekhlafi**<br>Abdulraoof **Malibary**<br>Beverly **Cuizon**<br>Hamzah **Ghazal**<br>Bodour Ayidh **Aldosari**<br>Basmah Alhumaidi **Alanazi**<br>Dania Abdullah **Mohanna**<br>Phil Noel **Conception**<br>Wafaa **Alokaili**<br>Abdullah Saleh **Al Shehri**<br>Tareef **Aldaghestani**<br>Czarina **Mahinay**<br>Najla **Al Mutairi**<br>Bander **Alanazi**<br>Adel **Altohary**<br>Sami Moshref **Alshehri**<br>Mohammad **Abdulmonem**<br>Riyadh **Alorf**
King Fahad Military Medical Complex, Dahran, Saudi Arabia	Abdulaziz Rashid **Al Shaer**<br>Khaled Abdulraheem **Al Faraidy**<br>Abdulrahman Abdullah **Al Ghamdi**<br>Mahmoud Hameed **Al Kurdi**<br>Mohammed Ibrahim **Al Othiqy**<br>Maryim Nasser **Al Dossari**<br>Reham Abdulrahman **Al Qahtani**<br>Sarah Abdrab Alrasool **Al Ghazal**<br>Sarah Hashim **Al Zahrani**<br>Ali Qasim **Ghazwani**
Armed Forces Hospital, Jazan, Saudi Arabia	Saud Mohammed **Erwi**<br>Ibrahim **Fadl**<br>Ahmad **Esaie**
Northern Area Armed Forces Hospital, Saudi Arabia	Mamdouh Abd **El hameed**<br>Mohammed **Shamea**
King Fahd Hospital of the University-Imam Abdulrahman Bin Faisal University, AlKhobar, Saudi Arabi	Mohammed Saeed **Alshahrani**<br>Yousef Ahmed **Almubarak**<br>Ashraf **Attia**

	Abdulaziz Saad **Alghamdi**<br>Hawra **Alnasir**<br>Laila Perlas **Asonto**<br>Charlene **Mapusao**<br>Thabit **Alotaibi**<br>Arivukodi **Muthu**
Royal Commission Health Services Program, Jubayl, Saudi Arabia	Abdullah **Albabtain**<br>Abdulrahman **Ismail**<br>Rashed Abdulrahman **Alqahtani**
