# Supplementary material for: Impact of a national collaborative project to improve the care of mechanically ventilated patients
Source: PLoS One. 2023 Jan 30;18(1):e0280744. doi: 10.1371/journal.pone.0280744 (PMC9886257; doi:10.1371/journal.pone.0280744)
Supplement: S2 Table — (PDF) [file pone.0280744.s002.pdf]

S2 Table: List of participating sites and ethics committee approvals for the National.

Site	Ethics Committee	Reference Number & Date of Approval
**Ministry of National Guard Health Affairs**		
King Abdulaziz Medical City, Riyadh	Institutional Review Board, National Guard Health Affairs	RC 17/223 21 December 2017
King Abdulaziz Medical City, Jeddah		
King Abdulaziz Hospital, Alhasa		
Prince Mohammed bin Abdulaziz Hospital, Madinah		
**Ministry of Health**		
King Saud Medical City, Riyadh,	Institutional Review Board	No. 18-431E 27 August 2018
Arar Central Hospital, Arar		
Alrass General Hospital, AlQassim		
King Fahad Hospital, Al Baha		
King Abdulaziz Specialist Hospital, Taif		
Gurayat General Hospital, AlGurayat		
Prince Mohammed Bin Abdulaziz Hospital, Skakka		
King Salman Hospital, Riyadh		
King Abdullah Hospital, Bisha		
King Khalid General Hospital, Majmaah		
Buraydah Central Hospital, AlQassim		
King Fahad Specialist Hospital, AlQassim		
King Faisal Hospital,Makkah		
King Khaled Hospital, Tabuk		
King Khalid Hospital, Hail		
King Khalid General Hospital, Hafer Al Batin		
Jubayl General Hospital, Jubayl		
King Khalid Hospital, Najran		
Qatif Central Hospital, Qatif		
Al Noor Specialist Hospital, Makkah		
Prince Mohammed Bin Abdulaziz Hospital, Riyadh		
King Fahad Hospital, AlMadinah		
King Fahad Specialist Hospital, Tabouk		
Dammam Medical Complex, Dammam		
King Faisal Medical Complex, Taif		
Prince Saud bin Jalawi, Al Hasa		
**King Faisal Specialist Hospital & Research Centre**		
King Faisal Specialist Hospital & Research Centre, Riyadh	Research Ethics Committee	RAC 2181-214 27 December 2018
King Faisal Specialist Hospital & Research Centre, Jeddah	Research Ethics Committee	RAC 2018-41 21 November 2018
**Military Medical Services**		
Prince Sultan Military Medical City, Riyadh	Scientific Research Center, Research Ethics Committee	No. 1135 14 November 2018
King Fahad Military medical complex, Dhahran		
Armed Forces Hospital, Jazan		
Northern Area Armed Forces Hospital		
**King Fahd Hospital of the University-Imam Abdulrahman**	Institutional Review	2018-01-323

**Bin Faisal University, AlKhobar**	Board	26 December 2018
**Royal Commission Health Services Program, Jubayl**	Research Center, Ethics Committee	No. 5-16-24<br>27 December 2018
