# Supplementary material for: Impact of a national collaborative project to improve the care of mechanically ventilated patients
Source: PLoS One. 2023 Jan 30;18(1):e0280744. doi: 10.1371/journal.pone.0280744 (PMC9886257; doi:10.1371/journal.pone.0280744)
Supplement: S3 Table — (PDF) [file pone.0280744.s003.pdf]

S3 Table: List of interventions.

Training Activities	
	Workshop
	Webinars
	Ad-hoc webinars
	Site visits
Science of safety topics covered in the workshop and webinars	
	The science of Patient Safety
	The concept of CUSP
	PDSA cycles
	Safety Culture
	Engagement of staff in patient safety
	Learning from defects
	Using data for improvement
	Sustainability
	Using daily goals during interdisciplinary rounds
Mechanical ventilation improvement topics	
	Opportunities for improving the care of mechanically ventilated patients
	NASAM bundle
	Early Mobility
	Daily Sedation interruption
	Spontaneous breathing trial
	VAE surveillance training
	Data Collection
	Daily goals
	Delirium: Assessment and prevention
	Subglottic endotracheal tubes
	Data collection
	Pain, agitation, Delirium (PAD), sedation management
	Low tidal volume ventilation
	Wake up and breathe
Online Resources	https://www.youtube.com/channel/UCOhXcUhxxJWt5GkTQNAUSqA/videos
