# Supplementary material for: Impact of a national collaborative project to improve the care of mechanically ventilated patients
Source: PLoS One. 2023 Jan 30;18(1):e0280744. doi: 10.1371/journal.pone.0280744 (PMC9886257; doi:10.1371/journal.pone.0280744)
Supplement: S4 Table — (PDF) [file pone.0280744.s004.pdf]

S4 Table: Data measures definitions.

Measure	Formula	Comment
Subglottic Suctioning Compliance Rate	= 100 X n of patient-days with subglottic suctioning/n of patients eligible for subglottic suctioning	Ineligible if subglottic suctioning is contraindicated due to tracheostomy or other reasons
Spontaneous Awakening Trial (SAT) Compliance Rate	= 100 X n of patient-days on SAT /n of patients eligible for SAT	Ineligible if SAT is contraindicated due to: increased intracranial pressure in the previous 24 hours, escalating sedative doses due to ongoing agitation, on neuromuscular blocker, sedatives for other seizures or objective evidence of alcohol withdrawal, high frequency oscillation ventilation, active myocardial ischemia in the previous 24 hours or others
Spontaneous Breathing Trials (SBT) Compliance Rate	= 100 X n of patient-days on SBT /n of patients eligible for SBT	Ineligible if SBT is contraindicated due to: no adequate oxygenation (SpO_2 <88% on an FiO_2 of 50% and PEEP of 8 cm H_2O), no spontaneous inspiratory effort in a 5-minute period, significant vasopressors or inotropes, increased intracranial pressure in the previous 24 hours, acute agitation requiring escalating sedative doses
Percentage of Actual RASS score (-1 to 1)	= 100 X n of patient-days with actual RASS score -1 or 0 or 1 /n of patients eligible for RASS score	-
Percentage of Achieving RASS Target	= 100 X n of patient-days with actual RASS score difference of -1 or 0 or 1 from the target RASS/n of patients eligible for RASS score	-
Percentage of ventilated patients receiving neuromuscular blocker	= 100 X n of patient-days on neuromuscular blocker /n of ventilated patients	-
Ventilator associated event (VAE) Rate	= 1000 X VAC + IVAC + PVAP / n of ventilator days	-
Ventilator associated condition (VAC) Rate	= 1000 X VAC / n of ventilator days	-
Infection related ventilator-associated complication (IVAC) Rate	= 1000 X IVAC / n of ventilator days	-
Possible ventilator associated pneumonia (PVAP) Rate	= 1000 X PVAP / n of ventilator days	-
ICU Mortality Rate	= % deaths among patients initiated on mechanical ventilation for the study month / n of ventilated patients	-

	for the study month	
Ventilator days	= n of ventilator days among patients initiated on mechanical ventilation for the study month / n of ventilated patients for the study month	-
Average ICU Length of Stay Per Patient	= n of ICU days among patients initiated on mechanical ventilation for the study month / n of ventilated patients for the study month	-
