# Supplementary material for: Impact of a national collaborative project to improve the care of mechanically ventilated patients
Source: PLoS One. 2023 Jan 30;18(1):e0280744. doi: 10.1371/journal.pone.0280744 (PMC9886257; doi:10.1371/journal.pone.0280744)
Supplement: S5 Table — IQR: interquartile range. (PDF) [file pone.0280744.s005.pdf]

S5 Table: Characteristics of participating sites.

	**N**
Number of ICUs	42
Number of ICU beds	1363
Number of hospital beds >300 (n, %)	29 (69)
Number of hospital beds ≤300 (n, %)	13 (31)
Number of physicians, Median (IQR)	8 (9)
Number of nurses, Median (IQR)	43 (63)
Number of respiratory therapists, Median (IQR)	8 (10)
Number of physiotherapists, Median (IQR)	2 (3)
Number of other allied health staff (pharmacists, dietician, etc), Median (IQR)	1 (3)
Duration of participation (months), median (IQR)	8 (7)

IQR: interquartile range
