# Supplementary material for: Impact of a national collaborative project to improve the care of mechanically ventilated patients
Source: PLoS One. 2023 Jan 30;18(1):e0280744. doi: 10.1371/journal.pone.0280744 (PMC9886257; doi:10.1371/journal.pone.0280744)
Supplement: S6 Table — (PDF) [file pone.0280744.s006.pdf]

S6 Table: Distribution of compliance, non-compliance and contraindications (with reasons) to the interventions.

**Spontaneous awakening trial (SAT) compliance**	**N**
Total observations	27929
SAT-Yes	9297
SAT-No	5310
Not sedated	9618
SAT contraindicated/not indicated	3704
**Reasons**	
Increased intracranial pressure in the previous 24 hours	420
Escalating sedative doses due to ongoing agitation	462
On neuromuscular blocker	259
Sedatives for other seizures or objective evidence of alcohol withdrawal	186
High frequency oscillation ventilation	109
Active myocardial ischemia in the previous 24 hours	29
Others	2239
**Spontaneous breathing trial (SBT) compliance**	**N**
Total observations	27967
SBT-Yes	13479
SBT-No	7756
SBT contraindicated/not indicated	6732
**Reasons**	
No adequate oxygenation (SpO2 <88% on an FiO2 of 50% and PEEP of 8cm H2O)	811
No spontaneous inspiratory effort in a 5-minute period	521
Significant vasopressors or inotropes	360
Increased intracranial pressure in the previous 24 hours	270
Acute agitation requiring escalating sedative doses	205
Others	2048
**Subglottic suctioning compliance**	**N**
Total observations	27967
Subglottic Suctioning -Yes	10486
Subglottic Suctioning -No	17354
Subglottic Suctioning contraindicated	127
**Reasons**	
Tracheostomy	106
Others	21
**Head of bed elevation**	**N**
Total observations	27967
Head of bed elevation -Yes	27380
Head of bed elevation -No	372
Head of bed elevation contraindicated	215
**Reasons**	
Cervical, thoracic or lumbar surgery or instability	104
Hypotension	36
Unstable physiologic status	25
Low cardiac index	8
Patient refusal	2
Others	40
