# Supplementary material for: Impact of a national collaborative project to improve the care of mechanically ventilated patients
Source: PLoS One. 2023 Jan 30;18(1):e0280744. doi: 10.1371/journal.pone.0280744 (PMC9886257; doi:10.1371/journal.pone.0280744)
Supplement: S1 Fig — (PDF) [file pone.0280744.s007.pdf]

S1 Figure*: Map of Saudi Arabia with distribution of participating ICUs. The size of each circle corresponds to the number of participating ICUs in each city.

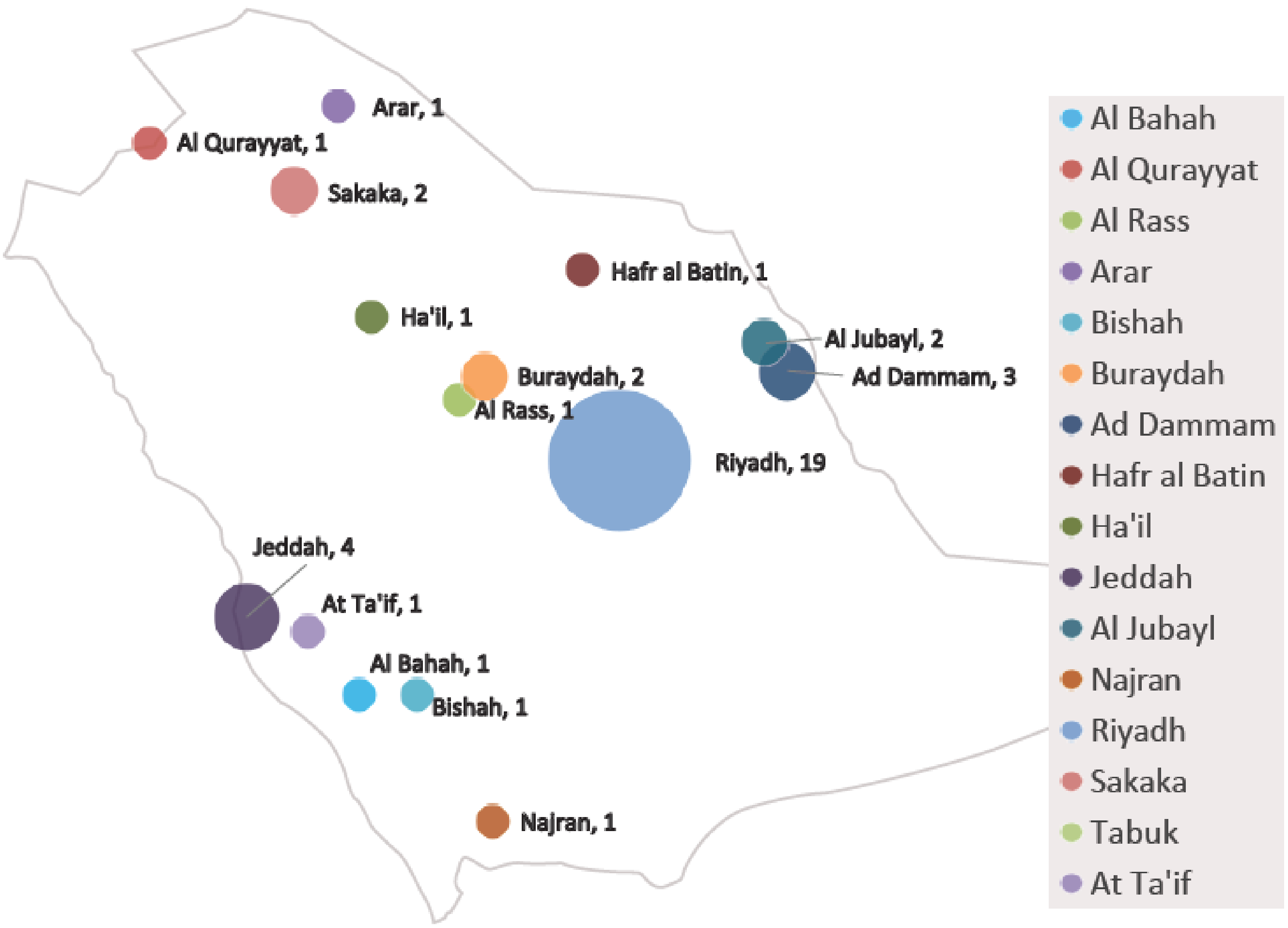

*The figure was drawn by an illustrator based on the official map of the Kingdom of Saudi Arabia (https://gasgi.gov.sa/Documents/Maps/2022/March/2m_Eng_10ED_02032022.pdf)
