# Supplementary material for: Impact of a national collaborative project to improve the care of mechanically ventilated patients
Source: PLoS One. 2023 Jan 30;18(1):e0280744. doi: 10.1371/journal.pone.0280744 (PMC9886257; doi:10.1371/journal.pone.0280744)

S3 Fig: Change of ICU mortality and ventilator-associated events (VAE) over the study period among the whole cohort and in subgroups of ICUs with baseline spontaneous trial (SAT) compliance of >50% and ≤50%.

All Patients	Baseline SAT >50	Baseline SAT≤50

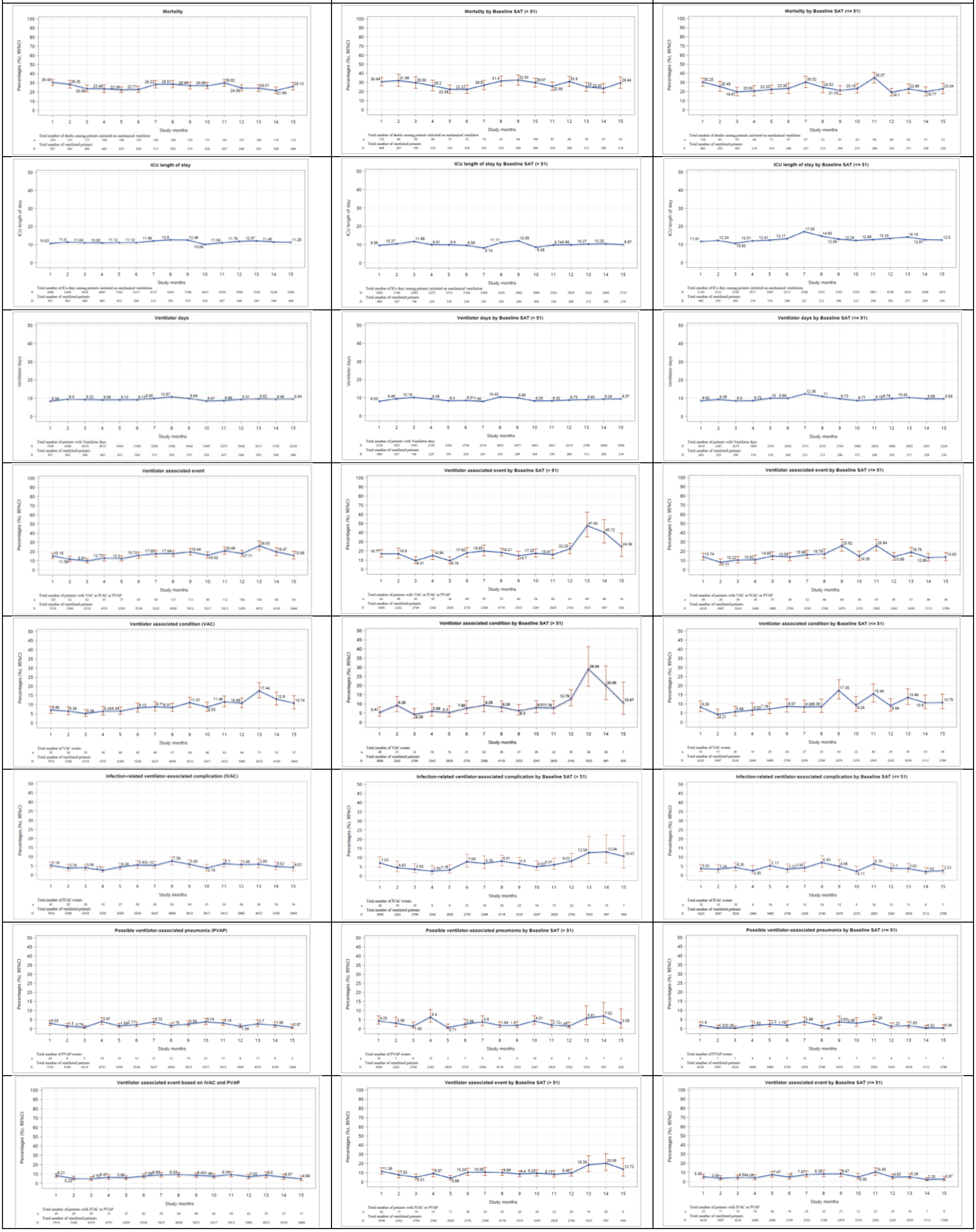

Supplement: S3 Fig — (PDF) [file pone.0280744.s009.pdf]
