# Supplementary material for: Impact of a national collaborative project to improve the care of mechanically ventilated patients
Source: PLoS One. 2023 Jan 30;18(1):e0280744. doi: 10.1371/journal.pone.0280744 (PMC9886257; doi:10.1371/journal.pone.0280744)
Supplement: S4 Fig — ** The random effect Poisson regression was used to estimate incidence rate ratio after incorporating ICU unit and hospital as random effects. ^^ The random effect negative binomial regression was used to estimate incidence rate ratio after incorporating ICU unit and hospital as random effects. IRR: Incidence rate ratio, CI: Confidence interval. (PDF) [file pone.0280744.s010.pdf]

S4 Fig: Forest plots for the change in subglottic suctioning, spontaneous breathing trial (SBT) and ventilator associated events (VAEs) in different subgroups. The p-value for interaction is shown.

Subgroup	n/N	IRR (95% CI) for monthly change	Pvalue	Pvalue for Interaction
**Subglottic suctioning compliance**				
Overall	10487/27840	1.035 (1.007, 1.064)	0.01	
ICUs with baseline SAT >51% (units=20)	5236/14123	1.038 (0.999, 1.079)	0.06	0.62^^
ICUs with baseline SAT≤51% (units=22)	5251/13717	1.032 (0.990, 1.076)	0.13	
ICUs with baseline SBT >61% (units=21)	5528/14793	1.038 (0.999, 1.079)	0.06	0.93^^
ICUs with baseline SBT ≤61% (units=21)	4959/13047	1.032 (0.990, 1.076)	0.13	
ICUs with baseline subglottic suctioning >25% (units=20)	8096/12889	0.997 (0.982, 1.012)	0.66	0.001^^
ICUs with baseline subglottic suctioning ≤25% (units=22)	2391/14951	1.116 (1.053, 1.183)	<0.001	
ICUs in hospitals >300 beds (units=29)	7072/21366	1.040 (1.005, 1.076)	0.03	0.60^^
ICUs in hospitals ≤300 beds (units=13)	3415/6474	1.020 (0.960, 1.084)	0.52	
**Spontaneous breathing trial (SBT) compliance**				
Overall	13479/21235	1.019 (1.009,1.029)	<0.001	
ICUs with baseline SAT >51% (units=20)	7796/9998	1.010 (0.997, 1.022)	0.12	0.12**
ICUs with baseline SAT≤51% (units=22)	5683/11237	1.032 (1.019, 1.045)		
ICUs with baseline SBT >61% (units=21)	7908/10342	1.010 (0.997, 1.022)	0.12	0.02**
ICUs with baseline SBT ≤61% (units=21)	5571/10893	1.032 (1.019, 1.045)		
ICUs with baseline subglottic suctioning >25% (units=20)	6514/9879	1.023 (1.012, 1.034)		0.66**
ICUs with baseline subglottic suctioning ≤25% (units=22)	6965/11356	1.015 (1.001, 1.030)	0.04	
ICUs in hospitals >300 beds (units=29)	9677/15422	1.019 (1.008, 1.030)	<0.001	0.72^^
ICUs in hospitals ≤300 beds (units=13)	3802/5813	1.019 (1.002, 1.037)	0.03	
**Ventilator associated events (VAE=VAC+IVAC+PVAC)**				
Overall	1350/83153	1.019 (0.985, 1.053)	0.28	
ICUs with baseline SAT >51% (units=20)	674/37981	1.012 (0.958, 1.069)	0.66	0.77**
ICUs with baseline SAT≤51% (units=22)	676/45172	1.023 (0.981, 1.066)	0.29	
ICUs with baseline SBT >61% (units=21)	638/37538	1.009 (0.954, 1.067)	0.75	0.64**
ICUs with baseline SBT ≤61% (units=21)	712/45615	1.025 (0.985, 1.067)	0.22	
ICUs with baseline subglottic suctioning >25% (units=20)	680/38305	1.026 (0.973, 1.082)	0.34	0.66**
ICUs with baseline subglottic suctioning ≤25% (units=22)	670/44848	1.011 (0.970, 1.053)	0.61	
ICUs in hospitals >300 beds (units=29)	831/68682	1.031 (0.996, 1.068)	0.08	0.45**
ICUs in hospitals ≤300 beds (units=13)	519/14471	0.995 (0.938, 1.057)	0.88	

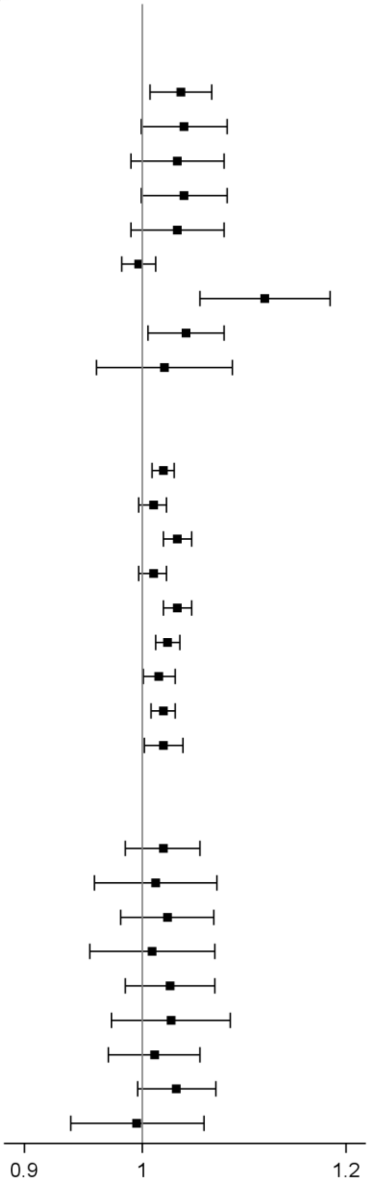

** The random effect Poisson regression was used to estimate incidence rate ratio after incorporating ICU unit and hospital as random effects.

^^ The random effect negative binomial regression was used to estimate incidence rate ratio after incorporating ICU unit and hospital as random effects.
IRR: Incidence rate ratio, CI: Confidence interval.
