# Supplementary material for: Impact of a national collaborative project to improve the care of mechanically ventilated patients
Source: PLoS One. 2023 Jan 30;18(1):e0280744. doi: 10.1371/journal.pone.0280744 (PMC9886257; doi:10.1371/journal.pone.0280744)
Supplement: S2 File — (PDF) [file pone.0280744.s012.pdf]

# National Approach to Standardize and Improve Mechanical Ventilation

# (NASAM)

المبادرة الوطنية لتحسين جودة الخدمات العلاجية لمرضى التنفس الاصطناعي (نسم)

**اسم المشروع:** المبادرة الوطنية لتحسين جودة الخدمات العلاجية لمرضى التنفس الاصطناعي (نسم)

وصف المشروع:

مشروع وطني عملي و تدريبي لتحسين جودة الخدمة العلاجية المقدمة لمرضى التنفس الصناعي في وحدات العناية المركزة في مستشفيات المملكة العربية السعودية للوصول الى المستوى المطلوب في تطبيق اليات التعامل مع مرضى اجهزة التنفس الصناعي حسب المعايير المبنية على البراهين و التعليم المستمر للطواقم الطبية.

أهداف المشروع:

1-ايجاد فرق عمل من الطواقم الطبية الموجودة في كل قسم عناية مركزة لتبني المشروع.
2- تدريب الطواقم الطبية على أليات التعامل مع مرضى التنفس الصناعي.
3- جمع معلومات عن الوضع الراهن و مدى تقيد الطواقم الطبية بالأليات.
4- اشراك جميع مستشفيات المملكة بالمشروع.

النتائج المتوقعة للمشروع:

1- تقليل نسبة الوفيات.
2- تقليل نسبة الاحداث و المخاطر الجسيمة الناتجة عن التنفس الصناعي
3- تقليل نسبة VAP (ذات الرئة)
4- تقليل نسبة الهدر المالي و البشري بسبب عدم تطبيق المعاير.

مؤشرات الأداء:

1- نسبة اشراك المستشفيات
2- التزام بحزمة معاير (نسم)
3- تقليل نسبة الامراض المتعلقة بالتنفس الصناعي
4- المؤشرات الحيوية بما فيها نسبة الوفيات

## A. Background

Several hospitals in Saudi Arabia have published findings on successful efforts to reduce Ventilator Associated Pneumonia (VAP), but these were mostly single center studies and focused on reducing VAP rather on an overall improvement in the care of mechanically ventilated patients.[1-8]

In 2013, the Armstrong Institute for Patient Safety and Quality at the Johns Hopkins Hospital, Maryland, USA, in collaboration with the Michigan Health & Hospital Association Keystone Center and the Department of Population Medicine at the Harvard Medical School and Harvard Pilgrim Health Care Institute (Massachusetts, USA), launched the Agency for Healthcare Research and Quality AHRQ-funded the Comprehensive Unit-Based Safety Program for improving the care of Mechanically Ventilated patients (CUSP 4 MVP project).[9] The objective was to improve the care of mechanically ventilated patients MVPs and eliminate preventable harm associated with mechanical ventilation and is run in 235 ICUs in 169 US hospitals.

A cohort of 15 ICUs from six hospitals in Saudi Arabia have participated this international effort to improve the care of mechanically ventilated patients from September 2015 to December 2016.[10] The project included multiple interventions (CUSP team formation, training, audit, and feedback) to improve several aspects of the care of mechanically ventilated patients following evidence-based guidelines. Over the relatively short period of time, there was significant improvement in key indicators of the care of mechanically ventilated patients with reduction in mortality. The cohort included 10124 patient days for 2634 patients. Over the intervention period, there was significant increase in the use of subglottic endotracheal tubes from 43% (663/1542) to 57% (287/505) $p<0.0001$, increase in days without sedation from 54% (3816/7017) to 71% (2197/3111) $p<0.0001$, and an associated reduction in mortality from 21% (372/1747) to 16% (147/887) $p=0.005$. There was no significant change in ventilator-associated events or the duration of mechanical ventilation. This project demonstrates the feasibility of performing a multicenter quality improvement project. A large-scale project with a longer duration may lead to major impact on the care of MVPs in Saudi Arabia.

In sustaining much of these efforts, we plan to extend the implementation to a total of 100 ICUs in collaboration with multiple health systems across the country. A significant component of this project includes measuring the safety culture of ICUs, which is one of the Ministry of Health indicators for Vision2030.[11]

## B. Objectives

NASAM is a national collaborative quality improvement project in Saudi Arabia which aims to:

1. Improve the care of mechanically ventilated patients
2. Reduce the rate of ventilator-associated pneumonia
3. Reduce risks and complications associated with mechanical ventilation

## C. Project Overview

Over the course of 2 years, ICU teams will join a network of hospitals in an effort to reduce VAP. These teams will be collecting VAP infection rates, attend online training sessions, and lead on local safety efforts to reduce patient harm. Participating teams are invited to join the NASAM project web portal. This portal provides educational materials for frontline staff, infection control practitioners and allied healthcare professionals; evidence-based toolkits; data-collection tools to evaluate local practices; and a robust web-based data platform to generate real-time data reports. Participating ICUs can track their performance over time and compare their performance with others. Reports can be shared with team members, frontline staff and hospital leaders to sustain engagement in the program.

## D. Benefits of Participating in a Study

1. Evaluation and comparison of local practices
2. a robust web-based data platform to generate real-time data reports.
3. Reports can be shared with team members, frontline staff and hospital leaders to sustain engagement in the program.

## E. Project Setting

We aim to recruit 100 ICUs across the country to participate in this 2 year quality improvement project. ICUs will be recruited using various methods: (1) registering their interest through The Saudi Critical Care Society and (2) contacting executives, ministers, and leaders for their support and participation.

**F. Design**

This is observational prospective project. Data will be collected from the ICUs and will be entered into the Ministry of National Guard-Health Affairs database. Data includes clinical (infection rates) and non-clinical data (safety culture survey results). The database will have the ability to generate reports based on the data entered into the system. Consent for participation is not required as the data will be de-identified.

**G. Interventions**

The project focuses on making changes through technical improvement and adaptive improvement.

**Technical Improvement**

1. **Data driven change:** The technical improvement involves using data to drive change. See Database section below.
2. **Education and training:** involves series of educational webinars that will be given biweekly on different aspects of the project. The topics will include the aspects listed in Table 1.

**Table 1:** Topics that will be covered in NASAM webinars.

| **Adaptive** | **Technical** |
|---|---|
| The science of Patient Safety | Opportunities for improving the care of mechanically ventilated patients |
| | |
| The concept of CUSP | NASAM bundle |
| PDSA cycles | Early Mobility |
| Safety Culture | Daily Sedation interruption |
| Engagement of staff in patient safety | Spontaneous breathing trial |
| Learning from defects | VAE surveillance training |
| Using data for improvement | Data Collection |
| Sustainability | Daily goals |
| Using daily goals during interdisciplinary rounds | Delirium: Assessment and prevention |
| | Subglottic endotracheal tubes |
| | Data collection |
| | Pain, agitation, Delirium (PAD), sedation |

| | |
|---|---|
| | management |
| | Low tidal volume ventilation |
| | Wake up and breathe |

3. **Coaching and audit and feedback**

**Adaptive Improvement**

This will be implemented using the concepts of the Comprehensive Unit-Based Safety Program (CUSP) to improve patient safety awareness and systems thinking at the unit level. Each unit will be asked to form a team (CUSP team) that meets monthly to discuss patient safety issues on the unit. CUSP allows frontline staff to partner with senior executives and leadership to collectively resolve patient safety issues. CUSP also offers tools to help teams learn from errors and improve teamwork and safety culture.

The CUSP approach was developed by patient-safety researchers at the Johns Hopkins Hospital (Baltimore, MD, USA).[12] CUSP is designed to improve local safety cultures and to guide to learn from mistakes by utilizing a structured framework.[13-15] This approach has been linked to large-scale reductions in healthcare-acquired infections,[16-19] mortality,[20] and associated costs.[21]

- **5 Steps of implementing a CUSP team:** [22] To implement the intervention, all participating units are requested to create a dedicated CUSP team. These teams ideally include a local physician and nursing champion, a senior executive, frontline healthcare providers (physicians, nurses, and ancillary staff), an infection control provider, and hospital quality and safety leaders.

  1. **Educate everyone in the "Science of Safety"**
     - Science of Safety is a one-hour session presenting system design, safe design principles, and valuing diverse input from clinical and non-clinical healthcare providers.
     - This includes: chairmen and department directors (the term "executives" will be used to identify leaders in upper-level management positions) AND clinical units
     - Identify CUSP team members: CUSP Coordinator/Facilitator, CUSP Champion, Nurse/Unit Manager, Physician Champion, Executive (chairmen/department director)

  2. **Identify defects**

- A defect is a clinical or operational event that you would not want to have happen again. These can be identified by administering the 2-Question Survey:
    1. How is the next patient likely to be harmed on our unit?
    2. What do you think we could do to prevent that harm?

**3. Recruit executives (chairmen and directors) as active CUSP team member**

- An executive is partnered with a CUSP team on a clinical unit to help address patient safety concerns and award patient safety successes

**4. Learn from one defect per quarter**

1. What happened?
2. Why did it happen? (Use system lenses from science of safety.)
3. What could you do to reduce risk ?
4. How do you know risk was reduced ?

**5. Implement teamwork tools**

- Culture surveys, handoff tools, shadowing, and other recommended tools

### H. Data Collection

Data collection is summarized in Table 2.

**Table 2:** Data Collection.

| Processes | Components | Requirements | Graphs Generated |
|---|---|---|---|
| NASAM Bundle | HOB ≥ $30^0$<br>Sedation at minimal level – sedation scale<br>Delirium assessment<br>SAT<br>SBT<br>Use of SUB-G ETT<br>Small tidal volume<br>Avoidance of neuromuscular blockers, unless there is a clear indication | Enter data daily or at least twice per week on all patients in your unit. | You will see your unit's compliance (blue line), compared to your cohort (green line), compared to the main cohort (black line) |
| Early Mobility | Current level of mobility<br>Identification of barriers to mobility<br>Clinical events associated with mobility<br>Delirium assessment | | |
| VAE Rates | VAC<br>iVAC<br>pVAP | Monthly data entry | |

| | |
|---|---|
| Objective | Mortality |
| Outcome | Length of stay |
| Measures | Duration of mechanical ventilation |

**NASAM Bundle**

1. Sub-glottic suctioning endotracheal tube usage;
2. Head of the bed elevation;
3. Management of sedation levels;
4. Spontaneous awakening and breathing trials
5. Small tidal volume
6. Avoidance of neuromuscular blockers, unless there is a clear indication

**Early Mobility**

1. Current level of mobility
2. Identification of barriers to mobility
3. Clinical events associated with mobility
4. Delirium assessment

**VAE Rates**

We will use the Center for Disease Control and Prevention (CDC) definitions of ventilator-associated events VAE.[23] The new algorithm uses objective criteria for the diagnosis of ventilator-associated conditions (VACs), and infection-related ventilator-associated complications (IVACs).[24] This approach thereby broadens the definition of harm suffered by ventilated patients beyond pneumonia to include pulmonary edema, atelectasis, and acute respiratory distress syndrome.[25] The concept of VAEs has been validated and shown to be associated with longer MV treatment duration and ICU and hospital stays, and higher mortality.[26-32]

**Components**

1. VAC (ventilator-associated condition);
2. iVAC (Infection-related Ventilator-Associated Complications);
3. pVAP (possible ventilator-associated pneumonia)

**Objective Outcome**

1. Mortality
2. Length of Stay (LOS)
3. Mechanical ventilation duration

**Patient Safety Culture Survey**

We will use the (AHRQ) funded the development of the Hospital Survey on Patient Safety Culture (HSOPSC). The survey includes 42 items that measure 12 composites of patient safety culture.[33] These domains are shown in Table 3.

**Table 3:** HSOPSC domains[33].

| Domains of HSPSC |
|---|
| 1. Communication openness |
| 2. Feedback and communication about error |
| 3. Frequency of events reported |
| 4. Handoffs and transitions |
| 5. Management support for patient safety |
| 6. Nonpunitive response to error |
| 7. Organizational learning—Continuous improvement |
| 8. Overall perceptions of patient safety |
| 9. Staffing |
| 10. Supervisor/manager expectations and actions promoting patient safety |
| 11. Teamwork across units |
| 12. Teamwork within units |

**Date sampling**

**Table 4.** Data sampling for patient level data.

| Measures | Frequency of data collection |
|---|---|
| NASAM Bundle | Twice a week |
| Early Mobility | Twice a week |
| VAE Rates | Monthly |
| Objective Outcome Measures | Monthly |
| safety culture | Yearly |

**For Survey Sample**

Hospital Survey on Patient Safety Culture (HSOPS) by AHRQ: All included ICU healthcare providers.

**I. NASAM Website:**

**Step 1:** Obtain IRB approval

Step 2. Once you have IRB approval contact nasam@ngha.med.sa to get access to the data.

**Step 2:** Start data entry for the processes as in the following Figures.

**Figure 1:** Homepage of NASAM.

**Figure 2:** Portal to different groups of variables.

**Figure 3:** Data entry view.

**Figure 4:** One of the data entry views.

**Figure 5:** Measures view: Real-time data visualization; which can be easily captured and shared by Print-screen or Printout.

**Figure 6:** VAE Rates form.

**Figure 7:** Objective Outcome Measures form.

## J. Ethical considerations

This project is a quality improvement project. There is no risk for subjects and there are no patient identifiers will be used. Therefore, consent is not required. IRB approvals will be requested from participating sites.

## K. Project Team

This project will be led coordinated by a Steering Committee with the following members:

**Table 5:** NASAM Steering committee.

| Organization | Steering Committee Members |
|---|---|
| The Ministry of National Guard-Health Affairs | Dr. Yaseen Arabi<br>Dr. Abdulmohsen Saawi<br>Mr. John Alchin<br>Fahad Hameed |
| The Ministry of Health | Dr. Zohair Al Aseri |
| The Saudi Critical Care Society | Dr. Yasser Mandourah<br>Dr. Amin Yousef |
| The Johns Hopkins Armstrong Institute for Patient Safety & Quality | Dr. Asad Latif<br>Dr. Sean Berenholtz |
| King Faisal Specialist Hospital & Research Center – Riyadh | Dr. Khalid Maghrabi |
| Assir Central Hospital – Abha | Dr. Ali Bshabshe |
| King Abdulaziz Medical City – Dammam | Dr. Mohammed Shahrani |
| King Abdulaziz Medical City – Madina | Dr. Aiman Kharaba |
| Other institutions | Will be added as per approvals |
| Project management | Mr. Abdullah Zahrani<br>Mr. Mohmmad Qarni<br>Ms. Eman Al Qasim<br>Ms. Navasha Singh |

**L. Work plan**

This prospective study consists of a 2- year project.

**Table 6:** Work plan.

| **Timeline and Milestones** | | | | | | | | | | | | |
|---|---|---|---|---|---|---|---|---|---|---|---|---|
| Month | 1-2 | 3-4 | 5-6 | 7-8 | 9-10 | 11-12 | 13-14 | 15-16 | 17-18 | 19-20 | 21-22 | 23-24 |
| Phase | | **Implementation** | | | | | | **Post-Implementation** | | | | |
| **Aim 1: Adapt and implement a multifaceted intervention to address VAP** | | | | | | | | | | | | |
| Assemble expert panel | x | | | | | | | | | | | |
| Review recommended practices | x | x | | | | | | | | | | |
| Develop VAP bundle | | x | x | | | | | | | | | |
| Gap analysis of existing practices | | | x | | | | | | | | | |
| Form CUSP teams | | x | x | | | | | | | | | |
| 2-day CUSP workshop | | | x | | | | | | | | | |
| Administer HSOPS survey | | | x | x | | | | | | | | |
| CUSP coaching webinars | | | | x | x | x | x | x | x | x | x | x |
| **Aim 2: Evaluate the impact of the multifaceted intervention on outcomes and assess the relationship of contextual factors** | | | | | | | | | | | | |
| Develop database and data collection tool | | x | x | | | | | | | | | |
| Data collection | | | | x | x | x | x | x | x | x | x | x |
| Data analysis | | | | | | x | | | x | | | x |

**Supplement: Intensive Care Unit Registration form**

Thank you for your interest in joining CUSP4MVP-VAP initiative. Kindly, complete the following information:

1. **Hospital information:**

   City : ____________________

   Name of the hospital: ____________________

   Bed Capacity of the Hospital: ____________________

   Type of the hospital:

   - ☐ National Guard Health affairs
   - ☐ Ministry of Health
   - ☐ Military Hospital
   - ☐ University Hospital
   - ☐ Security Forces Hospital
   - ☐ King Faisal Specialist Hospital & Research Center
   - ☐ Private hospital
   - ☐ Other :____________________

2. **Intensive Care Unit information:**

   Name of the ICU:

   Bed Capacity in the ICU:

   Type of the ICU:

   - ☐ Medical
   - ☐ Surgical

**References:**


1. Arabi Y, Al-Shirawi N, Memish Z, Anzueto A. Ventilator-associated pneumonia in adults in developing countries: a systematic review. *Int J Infect Dis* 2008; **12**(5): 505-12.
2. Al-Tawfiq JA, Amalraj A, Memish ZA. Reduction and surveillance of device-associated infections in adult intensive care units at a Saudi Arabian hospital, 2004-2011. *Int J Infect Dis* 2013; **17**(12): e1207-11.
3. Al-Thaqafy MS, El-Saed A, Arabi YM, Balkhy HH. Association of compliance of ventilator bundle with incidence of ventilator-associated pneumonia and ventilator utilization among critical patients over 4 years. *Ann Thorac Med* 2014; **9**(4): 221-6.
4. Al-Dorzi HM, El-Saed A, Rishu AH, Balkhy HH, Memish ZA, Arabi YM. The results of a 6-year epidemiologic surveillance for ventilator-associated pneumonia at a tertiary care intensive care unit in Saudi Arabia. *Am J Infect Control* 2012; **40**(9): 794-9.
5. Al-Tawfiq JA, Abed MS. Decreasing ventilator-associated pneumonia in adult intensive care units using the Institute for Healthcare Improvement bundle. *Am J Infect Control* 2010; **38**(7): 552-6.
6. El Azab SR ESA, Abdelkarim M, Al Mutairi, KB, Al Saqabi A, El Demerdash, S. Combination of ventilator care bundle and regular oral care with chlorhexidine was associated with reduction in ventilator associated pneumonia. *Egyptian Journal of Anaesthesia* 2013; **29**: 273–7.
7. Garout M. Compliance and Association of Ventilator Associated Pneumonia Bundle Strategy With Ventilator Associated Pneumonia rate: A Saudi Experience. *The Journal of Bahria University Medical and Dental College* 2013; **3**(3): 11-5.
8. Khan R, Al-Dorzi HM, Al-Attas K, et al. The impact of implementing multifaceted interventions on the prevention of ventilator-associated pneumonia. *Am J Infect Control* 2016; **44**(3): 320-6.
9. Armstrong Institute for Patient Safety and Quality. CUSP for Mechanically Ventilated Patients - Ventilator Associated Pneumonia (CUSP 4 MVP-VAP). https://armstrongresearch.hopkinsmedicine.org/cusp4mvp.aspx.
10. The International Council for Harmonisation of Technical Requirements for Pharmaceuticals for Human Use (ICH): http://www.ich.org/fileadmin/Public_Web_Site/ICH_Products/Guidelines/Efficacy/E9/Step4/E9_Guideline.pdf. 5 February 1998. Last accessed Oct 29, 2017.
11. Edrees H, Al Aseri Z, Mandourah Y, et al. Commitment to collaborate: The value of establishing multicenter quality improvement collaboratives in Saudi Arabia. *Saudi Critical Care Journal* 2017; **1**(6): 7-9.
12. CUSP Toolkit. http://www.ahrq.gov/professionals/education/curriculum-tools/cusptoolkit/index.html (accessed 4/12/2015.
13. Vigorito MC, McNicoll L, Adams L, Sexton B. Improving safety culture results in Rhode Island ICUs: lessons learned from the development of action-oriented plans. *Jt Comm J Qual Patient Saf* 2011; **37**(11): 509-14.
14. Weaver SJ, Lofthus J, Sawyer M, et al. A Collaborative Learning Network Approach to Improvement: The CUSP Learning Network. *Jt Comm J Qual Patient Saf* 2015; **41**(4): 147-59.
15. Pronovost PJ, Berenholtz SM, Goeschel CA, et al. Creating high reliability in health care organizations. *Health Serv Res* 2006; **41**(4 Pt 2): 1599-617.
16. Pronovost P, Needham D, Berenholtz S, et al. An intervention to decrease catheter-related bloodstream infections in the ICU. *N Engl J Med* 2006; **355**(26): 2725-32.
17. Berenholtz SM, Lubomski LH, Weeks K, et al. Eliminating central line-associated bloodstream infections: a national patient safety imperative. *Infect Control Hosp Epidemiol* 2014; **35**(1): 56-62.
18. Berenholtz SM, Pham JC, Thompson DA, et al. Collaborative cohort study of an intervention to reduce ventilator-associated pneumonia in the intensive care unit. *Infect Control Hosp Epidemiol* 2011; **32**(4): 305-14.
19. Latif A, Kelly B, Edrees H, et al. Implementing a multifaceted intervention to decrease central line-associated bloodstream infections in SEHA (Abu Dhabi Health Services Company) intensive care units: the Abu Dhabi experience. *Infect Control Hosp Epidemiol* 2015; **36**(7): 816-22.

20. Lipitz-Snyderman A, Steinwachs D, Needham DM, Colantuoni E, Morlock LL, Pronovost PJ. Impact of a statewide intensive care unit quality improvement initiative on hospital mortality and length of stay: retrospective comparative analysis. *BMJ* 2011; **342**: d219.
21. Waters HR, Korn R, Jr., Colantuoni E, et al. The business case for quality: economic analysis of the Michigan Keystone Patient Safety Program in ICUs. *Am J Med Qual* 2011; **26**(5): 333-9.
22. AHRQ's Healthcare-Associated Infections Program. http://www.ahrq.gov/professionals/quality-patient-safety/hais/index.html (accessed 7/12/2015.
23. Prevention CfDCa. Ventilator-Associated Event (VAE). http://www.cdc.gov/nhsn/PDFs/pscManual/10-VAE_FINAL.pdf (accessed 30/10/2015.
24. Ventilator-AssociatedEvent(VAE). http://www.cdc.gov/nhsn/PDFs/pscManual/10-VAE_FINAL.pdf (accessed 6/1/2016.
25. Klompas M. Complications of mechanical ventilation--the CDC's new surveillance paradigm. *N Engl J Med* 2013; **368**(16): 1472-5.
26. Klompas M, Kleinman K, Murphy MV. Descriptive epidemiology and attributable morbidity of ventilator-associated events. *Infect Control Hosp Epidemiol* 2014; **35**(5): 502-10.
27. Zhu S, Cai L, Ma C, et al. The Clinical Impact of Ventilator-Associated Events: A Prospective Multi-Center Surveillance Study. *Infect Control Hosp Epidemiol* 2015; **36**(12): 1388-95.
28. Klompas M, Khan Y, Kleinman K, et al. Multicenter evaluation of a novel surveillance paradigm for complications of mechanical ventilation. *PLoS One* 2011; **6**(3): e18062.
29. Klompas M, Kleinman K, Khan Y, et al. Rapid and reproducible surveillance for ventilator-associated pneumonia. *Clin Infect Dis* 2012; **54**(3): 370-7.
30. Prospero E, Illuminati D, Marigliano A, et al. Learning from Galileo: ventilator-associated pneumonia surveillance. *Am J Respir Crit Care Med* 2012; **186**(12): 1308-9.
31. Hayashi Y, Morisawa K, Klompas M, et al. Toward improved surveillance: the impact of ventilator-associated complications on length of stay and antibiotic use in patients in intensive care units. *Clin Infect Dis* 2013; **56**(4): 471-7.
32. Klompas M, Anderson D, Trick W, et al. The preventability of ventilator-associated events. The CDC Prevention Epicenters Wake Up and Breathe Collaborative. *Am J Respir Crit Care Med* 2015; **191**(3): 292-301.
33. *Famolaro T, Yount N, Hare, R, et al Hospital Survey on Patient Safety Culture 2018 User Database Report (Prepared by Westat, Rockville, MD, under Contract No HHSA 290201300003C) Rockville, MD: Agency for Healthcare Research and Quality; 2018 AHRQ Publication No 18-0025-EF*.
